# Activity of New Synthetic (2-Chloroethylthio)-1,4-naphthoquinones in Prostate Cancer Cells

**DOI:** 10.3390/ph14100949

**Published:** 2021-09-22

**Authors:** Sergey A. Dyshlovoy, Dmitry N. Pelageev, Lea S. Jakob, Ksenia L. Borisova, Jessica Hauschild, Tobias Busenbender, Moritz Kaune, Ekaterina A. Khmelevskaya, Markus Graefen, Carsten Bokemeyer, Victor Ph. Anufriev, Gunhild von Amsberg

**Affiliations:** 1 Laboratory of Experimental Oncology, Department of Oncology, Hematology and Bone Marrow Transplantation with Section Pneumology, Hubertus Wald-Tumorzentrum, University Medical Center Hamburg-Eppendorf, Martinistrasse 52, 20251 Hamburg, Germany; lea.jakob@stud.uke.uni-hamburg.de (L.S.J.); j.hauschild@uke.de (J.H.); tobias.busenbender@stud.uke.uni-hamburg.de (T.B.); moritz.kaune@stud.uke.uni-hamburg.de (M.K.); c.bokemeyer@uke.de (C.B.); g.von-amsberg@uke.de (G.v.A.); 2 Martini-Klinik, Prostate Cancer Center, University Hospital Hamburg-Eppendorf, Martinistrasse 52, 20251 Hamburg, Germany; graefen@martini-klinik.de; 3 School of Natural Sciences, FEFU Campus, Far Eastern Federal University, Ajax Bay 10, Russky Island, 690922 Vladivostok, Russia; pelageev@mail.ru (D.N.P.); khea-96@mail.ru (E.A.K.); 4 G.B. Elyakov Pacific Institute of Bioorganic Chemistry, Far-East Branch, Russian Academy of Sciences, 690022 Vladivostok, Russia; borisovaksenia@mail.ru (K.L.B.); anufriev@piboc.dvo.ru (V.P.A.)

**Keywords:** 1,4-naphthoquinone, 2-chloroethylthio, castration-resistant prostate cancer, ROS, apoptosis, mitochondria

## Abstract

Development of resistance to currently available standard therapies in advanced prostate cancer (PCa) emphasizes the need for novel therapeutic options. Here, we report the synthesis of new hybrid molecules consisting of 2-chloroethylthio and 1,4-naphthoquinone pharmacophores and describe their activity in PCa. In screening analyses, the introduction of one 2-chloroethylthio group improved the anticancer properties of 1,4-naphthoquinones, whereas the introduction of a second 2-chloroethylthio moiety rather decreased activity. Two most promising of the synthesized compounds, **30** and **32**, were highly active in different human PCa cell lines harboring varying resistance profiles at nanomolar concentrations. The generated data suggest that the compounds are capable of mitochondria targeting, cytotoxic ROS induction, and DNA damage, which resulted in apoptosis presumably executed in a caspase-dependent manner. The substances synergized with the clinically approved PARP inhibitor olaparib and resensitized AR-V7-expressing PCa cells to antiandrogen enzalutamide, as well as to a combination of enzalutamide and an AKT inhibitor. This was at least in part exerted via down-regulation of AR-V7 expression and inhibition of AR signaling. Mild antagonism was observed in combination with platinum- or taxane-based chemotherapy, which was putatively related to treatment-induced activation of p38, JNK1/2, ERK1/2, MEK1/2, and AKT, functioning as potential pro-survival factors. Thus, the synthesized (2-chloroethylthio)-1,4-naphthoquinone derivatives exhibit promising anticancer properties in vitro, suggesting their further development as potential therapeutics for the treatment of castration-resistant PCa.

## 1. Introduction

Tremendous progress in the treatment of advanced prostate cancer (PCa) has resulted in increased overall survival and quality of life of the patients. At the same time, the development of resistance to current medications remains a key challenge in the course of disease. Androgen receptor targeting agents (ARTA), e.g., abiraterone, apalutamide, and enzalutamide, have proven efficacy in the androgen-dependent and androgen-independent stages of disease. However, in disease progression, there is an increasing loss of hormone dependence, which is mediated by mutations, splice variants, amplifications or a complete loss of the androgen receptor (AR), among others. In fact, a decreased activity of the second-generation ARTA has been reported in different clinical trials [1]. In this situation, chemotherapy-based treatment approaches continue to be of use. However, loss of taxane efficacy has been reported as well in advanced treatment lines [2,3,4]. Hence, new effective anticancer medications capable of overcoming drug resistance to standard medications are urgently needed.

In the past decades, new therapeutic options have become available for patients suffering from cancer and cancer-related conditions. Most of these novel medications target “classical” cancer-related proteins and processes, such as metabolism of fast-proliferating cells (e.g., 5-fluorouracil [5]), kinases, and other key regulators of signal transduction (e.g., imatinib, vemurafenib, and abemaciclib [6]), DNA repair defects (olaparib, niraparib, and AZD1390 [7]), and growth receptors and surface antigens (e.g., cetuximab and pertuzumab [8]). In drug development, improvement of pharmacological properties can be achieved by modification of the active molecules, e.g., by conjugation with a cargo molecule or pharmacophore. Examples of such modifications are antibody–drug conjugates (ADC). ADC provide specific delivery of cytotoxic agents to cancer cells expressing the target protein the antibody is directed to (e.g., enfortumab vedotin [8]). In addition, radioimmune conjugates (conjugates of an antibody to a chelated radioactive element) as well as conjugates of two or even three drug molecules have been developed. The conjugation of several pharmacophores (drugs), or so-called drug–drug conjugates, form a promising novel group of drugs in therapeutic development. Such hybrid molecules are capable of simultaneously targeting several biological processes and therefore increase therapeutic efficacy. To date, most of the drug conjugates approved for clinical applications are ADC. However, an increasing number of other drug–drug constructs have been synthesized and actively studied, such as conjugates of the CDK4 inhibitor ribociclib with the HDAC inhibitor vorinostat, the PARP inhibitor olaparib with vorinostat, the DNA-alkylating agent aniline mustard with the selective estrogen receptor modulator (SERM) combretastatin, or the antimitotic compound endoxifen with combretastatin, and many others [9]. Furthermore, several drug conjugates with AR ligands have been synthesized and are currently in pre-clinical testing in prostate cancer. Among them, there are cisplatin conjugated with ethisterone (testosterone homolog), doxaliform hybrid with alkyl-cyano nilutamide, and others [10].

Cancer cells are rapidly proliferating cells; therefore DNA and DNA-related processes play a key role in tumor progression. In the current study, we combined several pharmacophores causing DNA damage in one drug molecule. We hypothesize that this approach improves the anticancer properties of our recently described and characterized “mother” compounds. Thus, our 1,4-naphthoquinone pharmacophores demonstrated promising activity in CRPC, harboring different levels of resistance to standard therapies [11,12]. In fact, the 1,4-naphthoquinone moiety is represented in different anticancer drugs already approved for clinical use, e.g., anthracycline antibiotics, dactinomycin, and mitomycin-C [13]. Potential mechanisms of action have been found to comprise the induction of reactive oxygen species (ROS) due to the formation of semiquinones and superoxide radicals, resulting in dsDNA breaks [13,14,15]. Additionally, p53-independent cancer cell death [16] and inhibition of topoisomerase II enzyme have been observed [17,18]. In our current research, we conjugated 1,4-naphthoquinone pharmacophores with alkylating moieties, i.e., 2-chloroethylthio group, to further improve cytotoxic activity toward cancer cells. Mono- and bis(2-chloroethyl)sulfide (also known as sulfur mustard) are cytotoxic agents which exert their biological action via alkylation of guanine nucleotides in the DNA and by provoking oxidative stress [19,20]. At the beginning of the 20th century, bis(2-chloroethyl)sulfide and its derivatives were developed as chemical weapons. However, later, their anticancer properties were discovered. This resulted in the development of the chemotherapeutic Mustargen^TM^ (mechlorethamine, chlormethine), widely used for the treatment of hematological malignancies, including leukemia and Hodgkin’s lymphoma [21,22]. Further modification of the drug molecule via its conjugation to estrogen derivative led to the development of Estracyt^TM^ (Emcyt^TM^, estramustine phosphate), which was used in the therapy of mCRPC alone or in combination with other drugs, e.g., docetaxel [23,24,25].

Here, we report the synthesis, anticancer activity, and mechanism of action of (2-chloroethylthio)-1,4-naphthoquinone hybrid molecules. Their anticancer activity (alone and in combination with established therapeutics) was studied in prostate cancer cells harboring different levels of castration resistance.

## 2. Results and Discussion

### 2.1. Synthesis of (2-Chloroethylthio)-1,4-naphthoquinon-2-yl Conjugates

The combination of two pharmacophores is a promising strategy to improve and optimize drug properties by combining the biomedical properties of the different “mother” structures. Previously, we have shown that the 1,4-naphthoquinone moiety possesses a promising anticancer activity via induction of cytotoxic ROS [12]. In the current research, we synthesized and analyzed hybrid molecules that contain both the 1,4-naphthoquinone core and a 2-chloroethylthio moiety (sulfur mustard), which targets DNA due to the irreversible alkylation of guanine residues [26]. Both effects are known to be cytotoxic and ultimately lead to apoptotic death of cancer cells. We hypothesized that the combination of these properties can further improve the cytotoxic activity of the hybrid molecule in PCa.

**Figure 1:**
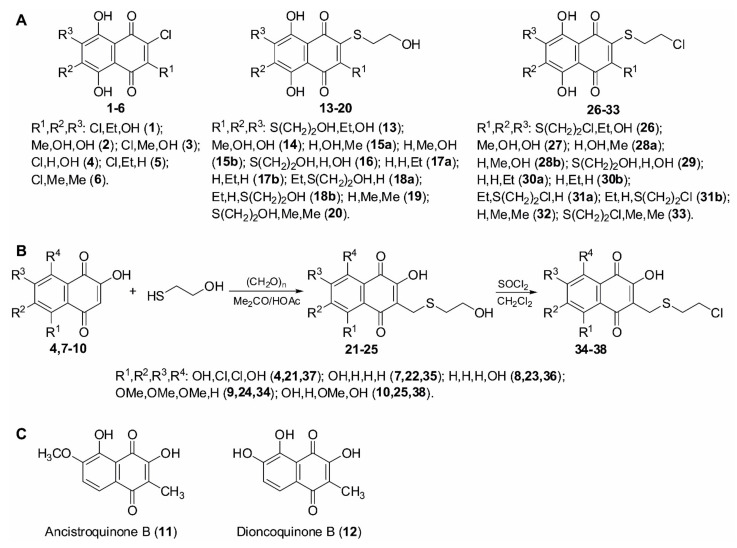
Synthesis of the investigated compounds. (**A**) Structures of the starting and synthesized compounds. (**B**) Scheme of synthesis. (**C**) Structures of ancistroquinone B and dioncoquinone B.

Thus, we have developed a synthetic approach to the novel 1,4-naphthoquinone derivatives which also contain 2-chloroethylthio moiety. Using this method, we have synthesized a small library of such compounds. For the synthesis, we used either (i) a substitution reaction of halogenoquinones (Figure 1A) or (ii) a thiomethylation reaction of 2-hydroxy-1,4-naphthoquinones (Figure 1B) with commercially available 2-mercaptoethanol. This was followed by the substitution of a hydroxy group with a chlorine atom under the action of SOCl_2_ in methylene chloride (Figure 1A,B).

### 2.2. Evaluation of Cytotoxic Activity and Selectivity of the Synthesized Compounds in Human Prostate Cancer Cells

The synthesized library was screened in human hormone-independent prostate cancer cells in order to identify the most promising candidates. Additionally, the two non-substituted quinones, namely ancistroquinone B (**11**) or dioncoquinone B (**12**) [27,28] (Figure 1C), were tested to determine the impact of the 2-chloroethylthio group introduction on biological activity. For efficacy screening, we selected androgen-independent 22Rv1 cell lines (AR-FL(+), AR-V7(+), BRCA2-mutated). Thus, we identified **30**, **31**, **32**, and **33** to be most active in these cells harboring IC_50_ values in low micromolar and nanomolar concentration ranges (Table 1). To identify the drug candidates which are most selective toward cancer cells, we also determined a cytotoxic activity in human prostate non-cancer PNT2 cells. Notably, in this experiment, the same four molecules exhibited the highest selectivity toward cancer cells and were therefore chosen for further investigations. In a next step, we further expanded the cell line panel and examined the cytotoxic activity of **30**, **31**, **32**, and **33** in five human prostate cancer and three human non-cancer cell lines to identify the most promising molecules.

During the progression of PCa, the tumor cells undergo specific epigenetic and genetic alterations, which ultimately result in therapy resistance and further progression. Here, loss of AR expression and AR mutations, as well as the expression of AR-V7, contribute to a hormone-independent phenotype and insensitivity to ARTA [29,30,31]. To evaluate the activity of the synthesized molecules in PCa cell lines bearing different AR status and therefore different levels of resistance to standard ARTA therapeutics, various PCa cell lines were used. Specifically, we included the androgen-independent PC-3 and DU145 cells (AR-FL(-), AR-V7(-)), which are also known to be rather resistant to taxanes; androgen-resistant 22Rv1 and VCaP cells (AR-FL(+), AR-V7(+)), which exhibit sensitivity to docetaxel and poor but still detectable sensitivity to antiandrogens due to the expression of both AR-FL and AR-V7; docetaxel-sensitive and androgen-dependent LNCaP cells (AR-FL(+), AR-V7(-)) (Table 2) [32,33,34,35]. Additionally, diverse non-cancer cell lines were used to examine toxicity in different types of non-malignant human cells (Table 2). Specifically, human epithelial PNT2 (prostate non-cancer) and HEK293 cells (non-prostate, non-cancer), as well as human fibroblasts MRC-9 (non-epithelial, non-prostate, non-cancer), were used (Table 2). Here, **30** and **32** showed the highest activity and selectivity and were therefore selected for further examinations.

Of note, **32** has been described to be an individual compound, whereas **30** appeared to be an equimolar and inseparable mixture of two regioisomers, namely 7-(2-chloroethylthio)-2-ethyl-5,8-dihydroxy-1,4-naphthoquinone (**30a**) and 6-(2-chloroethylthio)-2-ethyl-5,8-dihydroxy-1,4-naphthoquinone (**30b**) (molar ratio 1:1; see Materials and Methods and Table S1.4).

Additionally, we analyzed structure–activity relationships (SAR) of the synthesized compounds. Based on the generated data, we conclude that introduction of the 2-chloroethylthio moiety to the 1,4-naphthoquinone core may improve the cytotoxic properties of 1,4-naphthoquinone derivatives; however, only some of those derivatives have exhibited a biological activity prior to this synthetic modification. Thus, the introduction of the 2-chloroethylthio group to non-cytotoxic ancistroquinone B (**11**) or dioncoquinone B (**12**) resulted in the non-cytotoxic product **28** (Figure 1 and Table 1). At the same time, the introduction of the 2-chloroethylthio group to the cytotoxic compound SAB-1 (previously reported by us in [11]) (Figure 1 and Table 1) led to a dramatic 5-fold increase in its activity in PC-3 cells (Table 2). Of note, while the introduction of one 2-chloroethylthio group resulted in a significant increase in the cytotoxic activity of the described 1,4-naphthoquinones, the introduction of a second 2-chloroethylthio group did not add additional activity (Figure 1 and Table 1). Thus, the cytotoxic activity of mono-2-chloroethylthio derivative **30** was comparable to the activity of bis(2-chloroethylthio) derivative **31** (Figure 1 and Table 1). Indeed, the addition of the second 2-chloroethylthio group in the neighbor position may even decrease cytotoxic activity, most likely due to steric reasons (e.g., 2-mono-(2-chloroethylthio) derivative **32** was more active in comparison to 2,3-bis(2-chloroethylthio) derivative **33**).

### 2.3. Induction of Caspase-Dependent Apoptosis by 30 and 32

**Figure 2:**
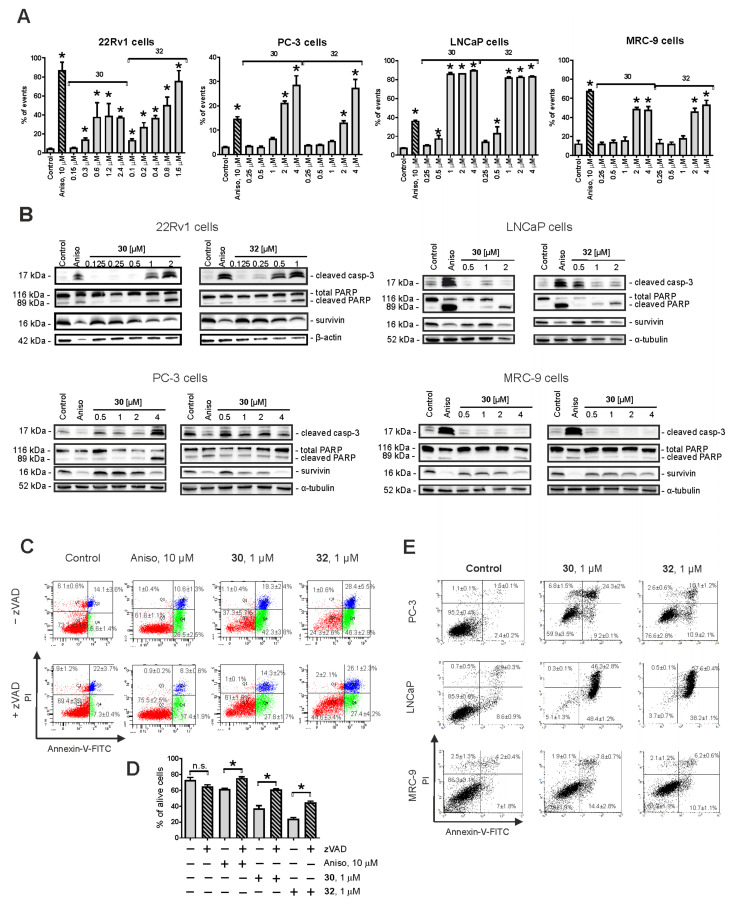
Pro-apoptotic activity of the synthesized compounds in 22Rv1, PC-3, LNCaP, and MRC-9 cells. (**A**) Analysis of drug-induced DNA fragmentation using PI staining and flow cytometry. Cells that appeared as sub-G1 population were assumed to be apoptotic and have been quantified with Cell Quest Pro software. (**B**) Analysis of the expression of pro-apoptotic proteins using Western blotting. β-Actin and α-tubulin were used as loading controls. (**C**–**E**) Analysis of apoptotic cells using annexin-V-FITC/PI double staining and flow cytometry. 22Rv1 cells were pre-treated with the pan-caspase inhibitor z-VAD(OMe)-fmk or vehicle (DMSO) for 1 h prior to treatment with the investigated drugs (**C**,**D**). PC-3, LNCaP, and MRC-9 cells were treated with the investigated drugs without pre-treatment with z-VAD(OMe)-fmk (**E**). Cells that appeared in the lower right and upper right quadrants were assumed to be apoptotic. The flow cytometry data were quantified with Cell Quest Pro software; double-negative cells (annexin-V^–^/PI^–^) were assumed as alive cells (**D**). Cells treated with 10 μM anisomycin (Aniso) were used as positive control. In all the experiments, the time of drug exposure was 48 h. Statistical significance: * *p* < 0.05 (Student’s *t*-Test, section **D**; or ANOVA followed by a post hoc Dunnett’s test, section **A**). n.s.–non significant (*p* > 0.05).

Next, the effects of highly effective and selective **30** and **32** on the apoptosis and proliferation of human PCa cells were examined. For this experiment, we used three PCa cell lines, namely, 22Rv1, PC-3, and LNCaP, with different AR status and thus different sensitivity to AR-targeting agents (see above). A dramatic dose-dependent DNA fragmentation was detected in all three cell lines following 48 h of drug treatment (Figure 2A). In addition, we found cleavage of caspase-3 and PARP in the treated cells (Figure 2B). Finally, externalization of phosphatidylserine to the outer membrane was detected in the cells exposed to the compounds **30** and **32** (Figure 2C–E). To investigate whether caspase-3 cleavage is an apoptosis-driving event and to exclude secondary effects of other cell death-related processes, we applied co-treatment with pan-caspase inhibitor z-VAD(OMe)-fmk (zVAD) in 22Rv1 cells (Figure 2C,D). In fact, pre-treatment with zVAD could significantly (however, not completely) inhibit apoptosis induced by both **30** and **32** (Figure 2C,D). Comparable effects were detected for anisomycin, a well-established inducer of classical apoptosis. An anti-apoptotic protein survivin was down-regulated by both compounds (Figure 2B). Of note, no significant effects on cell cycle progression were detected in either cell line (data not shown), suggesting a predominantly cytotoxic and not cytostatic activity of the compounds. Remarkably, the pro-apoptotic effects of both **30** and **32** were less pronounced in non-cancer MRC-9 cells, underlining their higher activity in cancer cells (Figure 2A–D). Taken together, both compounds exhibit promising anticancer activity exerted via induction of DNA damage and at least in part via classical caspase-dependent apoptosis.

For further studies of the mechanism of action, we used androgen-independent 22Rv1 cells (AR-FL( + ), AR-V7( + ), BRCA2-mutated). This model was selected as it allows us to study, among others, the effect on hormone therapy resistance caused by the expression of clinically relevant AR-V7, as well as the effect on AR signaling.

### 2.4. Mitochondria Targeting

**Figure 3:**
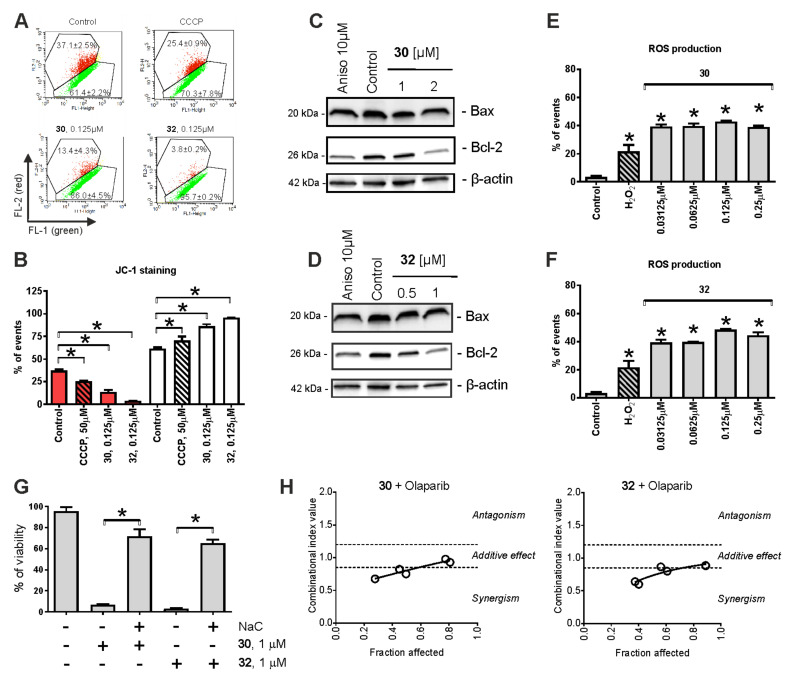
Effect of **30** and **32** on mitochondria of prostate cancer cells. (**A**,**B**) The induction of ΔΨm loss (mitochondrial membrane potential) by the drugs. 22Rv1 cells were treated for 2 h with the tested compounds and stained with JC-1, followed by flow cytometry analysis (**A**) and data quantification (**B**). The results indicate a drop in red fluorescence intensity, suggesting drug-induced depolarization of mitochondria. Cells showing ΔΨm loss have been quantified using Cell Quest Pro software (**B**). Cells treated with 50 μM CCCP for the indicated time were used as a positive control. (**C**,**D**) Western blotting analysis of Bax and Bcl-2 expression in 22Rv1 cells following 48 h drug exposure. (**E**,**F**) Effects on the ROS level in 22Rv1 cells. The cells were incubated with CM-H_2_DCFDA, followed by treatment with the investigated drugs for 2 h. The cells were harvested and analyzed by flow cytometry. H_2_O_2_-treated cells were used as a positive control. (**G**) Effect of N-acetyl-L-cysteine (NaC) on the cytotoxicity of **30** and **32**. 22Rv1 cells were pre-treated with 1 mM NaC for 1 h and then the investigated drugs were added to FBS- and glucose-free RPMI media. Cell viability was measured by the MTT test following 48 h of incubation with the drugs. (**H**) Effect of **30** or **32** in combination with olaparib. Cell viability was evaluated using the MTT assay and the data were analyzed using CampuSyn 1.0 software. Statistical significance: * *p* < 0.05 (Student’s *t*-test, section **G**; or ANOVA followed by a post hoc Dunnett’s test, sections **B**, **E** and **F**).

Previously, we reported that 1,4-naphthoquinones and their derivatives may target mitochondria in cancer cells [12]. Therefore, we assessed the effects of **30** and **32** on the mitochondrial membrane potential (ΔΨm) using the JC-1 staining and flow cytometry techniques (Figure 3A,B). Indeed, both compounds, as well as the well-established mitochondrial oxidative phosphorylation uncoupler CCCP, provoked mitochondrial membrane depolarization (ΔΨm drop down), suggesting mitochondria targeting by the synthesized derivatives (Figure 3A,B). Additionally, we observed down-regulation of Bcl-2 expression (Figure 3C,D), which may also contribute to the ΔΨm loss. Note that no significant changes in Bax expression level were detected (Figure 3C,D).

Elevated ROS levels are known to be associated with the disruption of mitochondrial integrity [36]. Therefore, the effects of **30** and **32** on ROS levels of 22Rv1 were evaluated using CM-H_2_DCFDA staining and further verified by flow cytometry analysis (Figure 3E,F). Indeed, the up-regulation of ROS was already observed after 2 h of treatment and at very low nanomolar concentrations of the drugs. Next, we determined the impact of ROS release on cell viability and its role in the cytotoxic activity of the investigated compounds. Therefore, PCa cells were pre-treated with an established antioxidant N-acetyl-L-cysteine (NaC), followed by the addition of **30** or **32** to the culture media and incubation for 48 h (Figure 3G). In fact, concurrent antioxidant therapy dramatically inhibited the cytotoxic effects of both compounds (Figure 3G), suggesting the drug-induced ROS release to have a major impact on the anticancer effects of **30** and **32**.

Generation of cytotoxic ROS, which may result from drug-induced damage of mitochondria, can further promote mitochondrial impairment [36]. Furthermore, ROS may damage DNA, which ultimately leads to apoptotic cell death [36]. Thus, increased ROS production causes single-strand DNA (ssDNA) breaks, which can be repaired by PARP, rescuing the cells from DNA-damage-induced apoptotic death [37,38]. In cells with insufficient ssDNA repair, double-strand breaks eventually occur. Homologous recombination repair mechanisms (HRR) come into play here. In HRR-deficient cells, however, double-strand breaks ultimately result in synthetic cell deaths [39,40,41]. Recently, the PARP inhibitor olaparib was approved for the treatment of mCRPC tumors, bearing germline deletions or somatic mutations of HRR genes, such as BRCA2 [39,42]. Hence, we examined the effects of **30** and **32** in combination with olaparib in 22Rv1 cells, known to bear mutated BRCA2 [42]. The experiments were conducted using the MTT viability test and the Chou–Talalay method. Indeed, combinational experiments revealed promising synergistic effects of **30** + olaparib as well as **32** + olaparib in 22Rv1 cells. These results emphasize the role of treatment-initiated cytotoxic ROS in drug-induced DNA damage and the consequent cell death, as well as indicate the combination of **30**/**32** with olaparib as a promising strategy for further drug development.

### 2.5. Cytotoxic Effects in Combination with Taxane and Platinum Agents

To identify additional, potential combination partners of 30 and 32, we evaluated the effect of the synthesized derivatives in combination with different therapeutics routinely used for the treatment of advanced PCa. Therefore, we co-treated PCa cells with 30 or 32 in combination with docetaxel. In addition, co-treatment with cisplatin and carboplatin was performed. The platinum agents are known to induce DNA cross-links and can be applied in the therapy of tumors harboring DNA repair defects or aggressive variants of PCa [42,43].

Of note, in combination with cisplatin and carboplatin, both 30 and 32 have revealed an additive to antagonistic effect, whereas in combination with docetaxel, a rather antagonistic effect was observed (Supplementary Materials, Figure S1). Therefore, in contrast to olaparib, a combination with these drugs does not seems to be feasible and should be avoided until further information on the potential drug interactions is available.

### 2.6. Effect on Stress-Activated Protein Kinases (SAPKs) and Other Kinases

**Figure 4:**
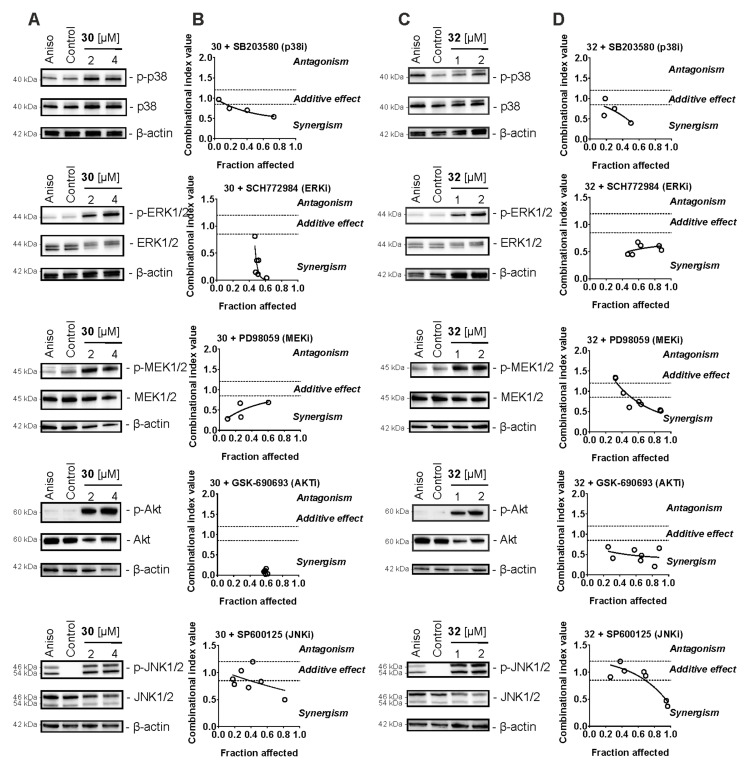
Analysis of the effect of compounds in combination with MAP kinases inhibitors (MAPKi). (**A**,**C**) 22Rv1 cells were treated with **30** (**A**) or **32** (**C**) for 2 h and the protein expression was analyzed by Western blotting. β-Actin was used as a loading control. Anisomycin (10 μM) was used as a positive control. (**B**,**D**) 22Rv1 cells were co-treated with **30**/**32** in combination with the JNK1/2 inhibitor SP600125, p38 inhibitor SB203580, MEK1/2 inhibitor PD98059, ERK1/2 inhibitor SCH772984, or AKT inhibitor GSK-690693 for 48 h. The viability was measured using the MTT assay and the effect of the drug combination was analyzed using CampuSyn 1.0 software.

Stress-activated protein kinases (SAPKs) play an important role in cellular response to stress conditions including chemotherapy exposure. An outcome of their activation can be both pro-cytotoxic and pro-survival, depending on the cell type and the nature of the stimulus [44]. It is known that mitochondrial stress and induction of ROS may result from activation of SAPKs [45]. Moreover, activation of some kinases such as JNK1/2 have been reported to mediate resistance to platinum-based therapies, taxanes, 5-FU, and other drugs [46]. Indeed, antagonism with cisplatin was observed in combination with 30 and 32 (see above). Therefore, we examined the effects of the synthesized compounds on stress-activated protein kinases JNK1/2 and p38, as well as the related mitogen-activated protein kinase (MAPK) ERK1/2, upstream MAPK kinase (MAPKK) MEK1/2, and AKT kinase. Following the 2 h treatment, we observed an activation of all of the above-listed kinases in 22Rv1 cells (Figure 4A,C). To further determine the impact of this broad kinase activation on the anticancer activity of 30 and 32, we applied combinational treatment with the corresponding specific kinase inhibitors (Figure 4B,D). Indeed, combinational treatment with inhibitors of SAPKs, namely p38i (SB203580) and JNKi (SP600125), revealed additive to synergistic effects of both inhibitors when combined with either 30 or 32 (Figure 4B,D). This suggests a treatment-induced activation of both p38 and JNK1/2, accompanied by decreased apoptotic effects of 30 and 32. In addition, we observed pronounced synergistic effects of both ERKi (SCH772984) and MEKi (PD98059) (Figure 4B,D). ERK1/2 is a direct and exclusive target of MEK1/2, and inhibition of MEK1/2 results in the inhibition of ERK1/2 [47]. Inhibition of this pathway increased the cytotoxic effect of both 30 and 32 (Figure 4B,D). Therefore, activation of the MEK/ERK pathway under treatment is a pro-survival factor and should be considered a resistance mechanism. Similar results were observed in combinational experiments with AKTi (GSK-690693) (Figure 4B,D). Of note, in this experiment, a very strong synergism was detected, suggesting the inhibition of AKT as a promising strategy to increase the cytotoxic activity of 30/32 (Figure 4B,D).

Therefore, the above-described slight antagonistic effects of **30**/**32** in combination with platinum and taxane agents can be at least partially explained by drug-induced activation of different stress kinases. Combination with pharmacological kinase inhibitors should be considered as a strategy in future pre-clinical and clinical trials to further improve the synthesized molecule efficacy.

### 2.7. Effect on AR Signaling

**Figure 5:**
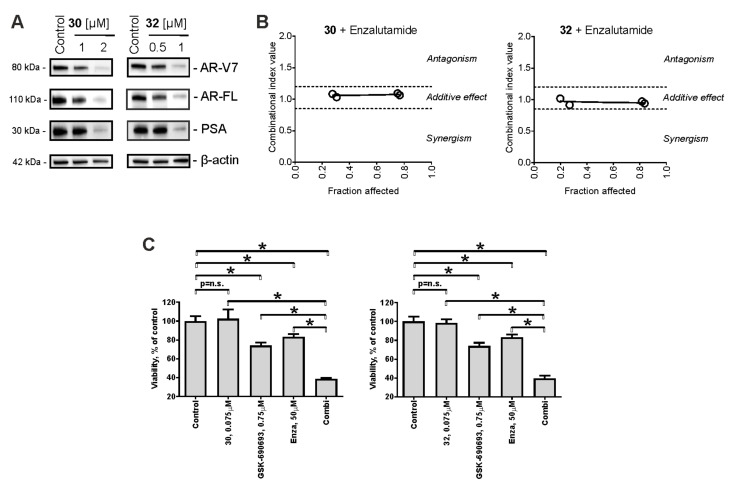
Analysis of the effect on AR signaling. (**A**) Effects of **30** and **32** on the expression of different proteins involved in AR signaling. Cells were treated for 48 h and the protein expression was accessed using Western blotting. β-Actin was used as a loading control. (**B**,**C**) 22Rv1 cells were co-treated with the synthesized compounds in combination with enzalutamide (**B**) or enzalutamide plus GSK-690693 (**C**) for 48 h. The viability was measured using the MTT assay. The effect of the combination of two drugs was calculated using CampuSyn 1.0 software (**B**). Statistical significance: * *p* < 0.05 (ANOVA followed by a post hoc Dunnett’s test).

Of note, a particular strong synergistic effect was observed for the synthesized drugs when combined with an AKT inhibitor (Figure 4B,D). An activated AKT pathway is known to mediate cell growth and proliferation in CRPC [48,49]. In fact, in prostate cancer therapy, the AKT pathway is often activated as a compensatory mechanism of AR pathway inhibition [49]. Therefore, we assumed that 30/32 may inhibit AR signaling in 22Rv1 cells, which then results in AKT activation. Hence, we examined the expression of PSA (downstream target of AR; often used to monitor AR transcriptional activity in vitro [50]) as well as AR—full length (AR-FL) in the cells following drug exposure. Of note, we identified the down-regulation of both proteins (Figure 5A), suggesting inhibition of AR signaling, presumably due to AR degradation. The AR signaling pathway is critical for the progression, proliferation, and survival of non-advanced human prostate cancer [29]. ARTAs suppress androgen production (e.g., abiraterone) or androgen binding to AR (e.g., enzalutamide) and have been proved to be highly active in the hormone-sensitive and castration-resistant stage [29]. However, development of resistance frequently occurs in the course of treatment accompanied by further tumor progression. An expression of AR-V7 has been reported to be a relevant ARTA-resistance mechanism [30,31]. AR-V7 lacks the C-terminal binding domain and therefore cannot bind to androgens or antiandrogens (i.e., enzalutamide) [29,30]. Moreover, AR-V7 autoactivates itself in the absence of androgens, resulting in a constantly active AR pathway, which leads to the promotion of PCa cell proliferation and survival [29,30]. 22Rv1 cells are known to express both full-length AR (AR-FL) as well as AR splice variant 7 (AR-V7). Hence, we further examined the effects of 30/32 on AR-V7 expression and observed a dose-dependent down-regulation of AR-V7 in the treated cells (Figure 5A). Thus, as the next step, the effect of 30/32 on enzalutamide activity in 22Rv1 was evaluated. In line with the above-reported results, a clear additive effect has been observed for both 30 and 32 when combined with enzalutamide (Figure 5B). Therefore, we speculate that 30/32 can inhibit AR signaling via inhibition of multiple AR isoforms, simultaneously sensitizing PCa cells to enzalutamide due to the inhibition of AR-V7 expression in particular. Currently, a Phase III clinical trial of a novel AKT inhibitor, ipatasertib, in combination with ARTA abiraterone is ongoing in patients suffering from mCRPC. As 30/32 resensitize PCa cells to enzalutamide, we hypothesized that the combination of 30/32 with an AKT inhibitor and an ARTA agent may also be beneficial. Hence, we treated 22Rv1 with the combination of GSK-690693 (ATKi), enzalutamide, and 30 or 32. In line with our assumption, we observed an increased cytotoxic activity of 30/32 plus GSK-690693 plus enzalutamide compared to the individual drugs (Figure 5C). Hence, such a drug combination should be considered a promising strategy for further studies and development of the synthesized hybrid molecules.

## 3. Materials and Methods

### 3.1. Chemistry

#### 3.1.1. General Synthetic Procedures and Methods

All melting points were determined with a Boetius apparatus (Dresden, Germany) and were uncorrected. Analytical grade chemicals and solvents were used. The ^1^H and ^13^C NMR spectra were recorded using Bruker Avance-300 (300 MHz), Bruker Avance III-500 HD (500 MHz) and Bruker Avance III-700 (700 MHz) spectrometers (Bruker Corporation, Germany) using CDCl_3_, acetone-d_6_, and DMSO-d_6_ as the solvents with the signal of the residual non-deuterated solvent as the internal reference. The IR absorption spectra were recorded using an IR-FT spectrophotometer, Bruker Equinox 55. Mass spectra were recorded using an Agilent 6510 Q-TOF LC mass spectrometer (Agilent, Santa Clara, CA, USA) (electrospray ionization, negative mode). The course of reactions was monitored by TLC. Merck Kieselgel 60F-254 plates (Kenilworth, NJ, USA) were preliminarily treated with 0.05 M tartaric acid in MeOH and dried at ~50 °C for 2–3 h; a 3:1 hexane/acetone mixture was used as an eluent. The purity of the compounds obtained was assessed by HPLC. Analytical HPLC of the samples was performed on a LaChrom (Merck Hitachi, Tokyo, Japan) system (pump L-7100, UV/ VIS detector L-7400, column oven L-7300, and integrator D-7500). Preparative column chromatography was performed on silica gel Alfa Aesar 40/65 μm (Ward Hill, MA, USA), using *n*-hexane-acetone. The yields were not optimized.

The following compounds have been described in the literature: **1–3**, **6** [51], **4** [52], **5** [53], **7, 8** [54], **9** [55], **10** [56], **11, 12** [57] (for details, see Table S1.1). The other investigated substances have been synthesized and purified as described below. The purity of the individual compound was verified by ^1^H and ^13^C NMR spectroscopy as well as HPLC. For details, please see the Supplementary Materials.

#### 3.1.2. General Procedure for the Synthesis of 2-Hydroxyethylthio Derivatives by Substitution of Chlorine Atoms in Chlorinated Naphthoquinones with 2-Mercaptoethanol in DMSO

To a solution of appropriate chlorinated naphthoquinone **1–6** (2 mmol) in DMSO (50 mL), 2-mercaptoethanol (780 mg, 10 mmol) and powdered potassium carbonate (138 mg, 1 mmol) were added. The reaction mixture was stirred for 10 h, diluted with water, acidified with dilute hydrochloric acid, and extracted with ethyl acetate. The extract was evaporated under reduced pressure and the solid was purified by column chromatography (silica gel, hexane-acetone, 5:1). For details, see Table S1.2.

2-Ethyl-3,5,8-trihydroxy-6,7-di(2-hydroxyethylthio)-1,4-naphthoquinone (**13**) obtained from 6,7-dichloro-2-ethyl-3,5,8-trihydroxy-1,4-naphthoquinone (**1**): dark violet solid; yield: 564 mg (73%); mp 142–146 °C. IR (CHCl_3_) ν_max_: 3419, 2937, 2878, 1601, 1382, 1365, 1330, 1279 cm^−1^. ^1^H NMR (CDCl_3_, 300 MHz): δ = 14.05 (s, 1 H, α-OH), 12.49 (s, 1 H, α-OH), 7.49 (br s, 1 H, β-OH), 3.71 (t, J = 5.0 Hz, 2 H), 3.67 (t, J = 5.0 Hz, 2 H), 3.47 (t, J = 5.0 Hz, 2 H), 3.34 (t, J = 5.0 Hz, 2 H), 2.63 (q, J = 7.3 Hz, 2 H), 1.15 (t, J = 7.3 Hz, 3 H). ^13^C NMR (CDCl_3_, 75 MHz): δ = 187.9, 181.2, 158.0, 157.9, 153.5, 143.8, 136.3, 127.0, 109.2, 108.8, 61.1, 60.6, 39.0, 38.7, 16.4, 12.5. HRMS (ESI): m/z [M-H]^−^ calcd for C_16_H_17_O_7_S_2_: 385.0421; found: 385.0424.

2,3,5,8-Tetrahydroxy-6-(2-hydroxyethylthio)-7-methyl-1,4-naphthoquinone (**14**) obtained from 6-chloro-2,3,5,8-tetrahydroxy-7-methyl-1,4-naphthoquinone (**2**): dark red solid; yield: 393 mg (63%); mp 195–200 °C.IR (KBr): 3480, 3402, 1677, 1658, 1599, 1451, 1406, 1303, 1279, 1218 cm^−1^. ^1^H NMR (acetone-d_6_, 300 MHz): δ = 13.08 (s, 1 H, α-OH), 12.68 (s, 1 H, α-OH), 9.03 (s, 2 H, β-OH), 3.86 (br s, 1 H, OH), 3.65 (t, J = 6.5 Hz, 2 H), 3.21 (t, J = 6.5 Hz, 2 H), 2.49 (s, 3 H, CH_3_). ^13^C NMR (DMSO-d_6_, 75 MHz): δ = 183.8, 183.4, 157.2, 155.0, 142.2, 141.7, 141.6, 135.1, 108.1, 107.1, 61.0, 36.8, 15.0. HRMS (ESI): m/z [M-H]^−^ calcd for C_13_H_11_O_7_S: 311.0231; found: 311.0232.

Mixture of 2,5,8-trihydroxy-6-(2-hydroxyethylthio)-3-methyl-1,4-naphthoquinone (**15a**) and 3,5,8-trihydroxy-6-(2-hydroxyethylthio)-2- methyl-1,4-naphthoquinone (**15b**) obtained from 6,7-dichloro-2,5,8-trihydroxy-3-methyl-1,4-naphthoquinone (**3**): dark red solid; yield: 326 mg (55%); HRMS (ESI): m/z [M-H]^−^ calcd for C_13_H_11_O_6_S: 295.0282; found: 295.0286.

2,5,8-Trihydroxy-6-(2-hydroxyethylthio)-3-methyl-1,4-naphthoquinone (**15a**). ^1^H NMR (CDCl_3_, 300 MHz): δ = 13.71 (s, 1 H, α-OH), 11.67 (s, 1 H, α-OH), 6.95 (s, 1 H), 3.96 (t, J = 6.0 Hz, 2 H), 3.22 (t, J = 6.0 Hz, 2 H), 2.09 (s, 3 H). ^13^C NMR (CDCl_3_, 75 MHz): δ =188.3, 179.1, 158.6, 155.6, 154.5, 145.6, 120.4, 119.5, 109.0, 107.1, 60.1, 33.9, 8.4.

3,5,8-trihydroxy-6-(2-hydroxyethylthio)-2-methyl-1,4-naphthoquinone (**15b**). ^1^H NMR (CDCl_3_, 300 MHz): ^1^H NMR (CDCl_3_, 300 MHz): δ = 12.92 (s, 1 H, α-OH), 12.16 (s, 1 H, α-OH), 7.08 (s, 1 H), 3.93 (t, J = 6.0 Hz, 2 H), 3.21 (t, J = 6.0 Hz, 2 H), 2.11 (s, 3 H). ^13^C NMR (CDCl_3_, 75 MHz): δ =186.7, 181.2, 158.1, 156.2, 153.1, 139.8, 124.5, 122.4, 109.2, 107.6, 60.1, 33.7, 8.2.

6,7-Di(2-hydroxyethylthio)-2,5,8-trihydroxy-1,4-naphthoquinone (**16**) obtained from 6,7-dichloro-2,5,8-trihydroxy-1,4-naphthoquinone (**4**): dark violet solid; yield: 437 mg (61%); mp 187–189 °C. IR (KBr): 3525, 2917, 1663, 1607, 1545, 1467, 1408, 1387, 1357 cm^−1^. ^1^H NMR (DMSO-d_6_, 300 MHz): δ = 13.91 (br s, 1 H, α-OH), 13.35 (br s, 1 H, α-OH), 12.27 (br s, 1 H, β-OH), 6.57 (s, 1 H), 5.65 (br s, 2 H, OH), 3.63–3.49 (m, 4 H), 2.75–2.63 (m, 4 H). ^13^C NMR (DMSO-d_6_, 75 MHz): δ = 186.1, 178.1, 162.3, 160.1, 158.4, 138.2, 136.5, 111.4, 110.5, 109.6, 61.5, 61.3, 36.9, 36.8. HRMS (ESI): m/z [M-H]^−^ calcd for C_14_H_13_O_7_S_2_: 357.0108; found: 357.0111.

Mixture of 2-ethyl-5,8-dihydroxy-7-(2-hydroxyethylthio)-1,4-naphthoquinone (**17a**) and 2-ethyl-5,8-dihydroxy-6-(2-hydroxyethylthio)-1,4-naphthoquinone (**17b**) obtained from 6,7-dichloro-2-ethyl-5,8-dihydroxy-1,4-naphthoquinone(5): red solid; yield: 265 mg (45%); HRMS (ESI): m/z [M-H]^−^ calcd for C_14_H_13_O_5_S: 293.0489; found: 293.0491.

2-Ethyl-5,8-dihydroxy-7-(2-hydroxyethylthio)-1,4-naphthoquinone (**17a**). ^1^H NMR (CDCl_3_, 300 MHz): δ = 12.75 (s, 1 H, α-OH), 12.64 (s, 1 H, α-OH), 7.07 (s, 1 H), 6.73 (s, 1 H), 3.92 (t, J = 6.0 Hz, 2 H), 3.12 (t, J = 6.0 Hz, 2 H), 2.72 (q, J = 7.5 Hz, 2 H), 1.91 (br s, 1H, OH), 1.25 (t, J = 7.5 Hz, 3 H). ^13^C NMR (CDCl_3_, 75 MHz): δ = 180.0, 179.7, 163.1, 162.7, 152.5, 147.0, 129.5, 127.0, 110.9, 109.4, 59.9, 33.5, 23.0, 12.6.

2-Ethyl-5,8-dihydroxy-6-(2-hydroxyethylthio)-1,4-naphthoquinone (**17b**). ^1^H NMR (CDCl_3_, 300 MHz): δ = 13.06 (s, 1 H, α-OH), 12.38 (s, 1 H, α-OH), 7.02 (s, 1 H), 6.72 (s, 1 H), 3.92 (t, J = 6.0 Hz, 2 H), 3.12 (t, J = 6.0 Hz, 2 H), 2.73 (q, J = 7.5 Hz, 2 H), 1.91 (br s, 1H, OH), 1.25 (t, J = 7.5 Hz, 3 H). ^13^C NMR (CDCl_3_, 75 MHz): δ = 180.8, 179.4, 163.5, 161.8, 153.2, 149.4, 127.3, 126.7, 110.3, 109.9, 59.9, 33.5, 23.1, 12.6.

Mixture of 3-ethyl-5,8-dihydroxy-2,6-di(2-hydroxyethylthio)-1,4-naphthoquinone (**18a**) and 2-ethyl-5,8-dihydroxy-3,6-di(2-hydroxyethylthio)-1,4-naphthoquinone (**18b**) obtained from 6,7-dichloro-2-ethyl-5,8-dihydroxy-1,4-naphthoquinone (**5**): red solid; yield: 200 mg (27%); HRMS (ESI): m/z [M-H]^−^ calcd for C_16_H_17_O_6_S_2_: 369.0472; found: 369.0474.

3-Ethyl-5,8-dihydroxy-2,6-di(2-hydroxyethylthio)-1,4-naphthoquinone (**18a**). ^1^H NMR (CDCl_3_, 300 MHz): δ = 13.23 (s, 1 H, α-OH), 13.08 (s, 1 H, α-OH), 6.82 (s, 1 H), 3.95 (t, J = 7.5 Hz, 2 H), 3.79 (t, J = 7.5 Hz, 2 H), 3.15 (t, J = 7.5 Hz, 2 H), 3.11 (t, J = 7.5 Hz, 2 H), 3.07 (q, J = 7.5 Hz, 2 H), 1.17 (t, J = 7.5 Hz, 3 H).

^13^C NMR (CDCl_3_, 75 MHz): δ = 175.6, 174.5, 167.9, 165.7, 154.2, 148.8, 136.1, 125.8, 110.1, 109.7, 60.7, 59.3, 36.1, 31.5, 22.8, 13.7.

2-Ethyl-5,8-dihydroxy-3,6-di(2-hydroxyethylthio)-1,4-naphthoquinone (**18b**). ^1^H NMR (CDCl_3_, 300 MHz): δ = 13.36 (s, 1 H, α-OH), 13.02 (s, 1 H, α-OH), 6.81 (s, 1 H), 3.93 (t, J = 7.5 Hz, 2 H), 3.75 (t, J = 7.5 Hz, 2 H), 3.17 (t, J = 7.5 Hz, 2 H), 3.13 (t, J = 7.5 Hz, 2 H), 3.06 (q, J = 7.5 Hz, 2 H), 1.16 (t, J = 7.5 Hz, 3 H).

^13^C NMR (CDCl_3_, 75 MHz): δ = 175.2, 174.1, 167.3, 167.1, 151.5, 149.1, 139.4, 125.3, 110.4, 109.0, 60.6, 59.4, 36.1, 31.5, 22.8, 13.7.

5,8-Dihydroxy-2-(2-hydroxyethylthio)-6,7-dimethyl-1,4-naphthoquinone (**19**) obtained from 2,3-dichloro-5,8-dihydroxy-6,7-dimethyl-1,4-naphthoquinone (**6**): purple solid; yield: 194 mg (33%); mp 133–137 °C. IR (CHCl_3_): 3662, 3610, 2929, 2883, 2857, 1715, 1600, 1552, 1432, 1391, 1302, 1261 cm^−1^. ^1^H NMR (CDCl_3_, 300 MHz): δ = 13.11 (s, 1 H, α-OH), 13.07 (s, 1 H, α-OH), 6.78 (s, 1 H), 3.97 (t, J = 6.0 Hz, 2 H), 3.15 (t, J = 6.0 Hz, 2 H), 2.25 (s, 3 H, Me), 2.24 (s, 3 H, Me). ^13^C NMR (CDCl_3_, 75 MHz): δ = 173.8, 172.6, 169.8, 168.5, 149.3, 141.9, 139.6, 125.1, 109.3, 108.4, 59.8, 33.5, 12.5, 12.3. HRMS (ESI): m/z [M-H]^−^ calcd for C_14_H_13_O_5_S: 293.0489; found: 293.0488.

5,8-dihydroxy-2,3-di(2-hydroxyethylthio)-6,7-dimethyl-1,4-naphthoquinone (**20**) obtained from 2,3-dichloro-5,8-dihydroxy-6,7-dimethyl-1,4-naphthoquinone (**6**): purple solid; yield: 356 mg (48%); mp 115–120 °C. IR (CHCl_3_): 2932, 2875, 1601, 1395, 1330, 1297, 1269 cm^−1^. ^1^H NMR (CDCl_3_, 300 MHz): δ = 13.50 (s, 2 H, 2 α-OH), 3.72 (t, J = 4.7 Hz, 4 H), 3.43 (t, J = 4.7 Hz, 4 H), 2.23 (s, 6 H, Me). ^13^C NMR (CDCl_3_, 75 MHz): δ = 172.7, 169.9, 144.3, 141.5, 109.6, 61.3, 38.7, 12.6. HRMS (ESI): m/z [M-H]^−^ calcd for C_16_H_17_O_6_S_2_: 369.0472; found: 369.0475.

#### 3.1.3. General Procedure for the Synthesis of (2-Hydroxyethylthio)methyl Derivatives by Acid-Catalytic Condensation of 2-Hydroxynaphthoquinones with 2-Mercaptoethanol and Paraformaldehyde in Acetone

To a solution of appropriate 2-hydroxy-1,4-naphthoquinone **4, 7–10** (1 mmol) in acetone (50 mL), 2-mercaptoethanol (156 mg, 2 mmol), acetic acid (0.20 mL), and paraformaldehyde powder (120 mg, 4 mmol) were added. The mixture was gently refluxed with stirring (2 h) until TLC indicated that the reaction was complete. The mixture was evaporated in vacuo and the solid was purified by column chromatography (silica gel, hexane-acetone, 5:1). For details, see Table S1.3.

6,7-Dichloro-2,5,8-trihydroxy-3-((2-hydroxyethylthio)methyl)-1,4-naphthoquinone (**21**) obtained from 6,7-dichloro-2,5,8-trihydroxy-1,4-naphthoquinone (**4**): red solid; yield: 347 mg (95%); mp 165–168 °C. IR (KBr): 3415, 1608, 1402, 1277, 1217, 1178, 1135, 1114, 1054, 990 cm^−1^. ^1^H NMR (DMSO-d_6_, 300 MHz): δ = 13.71 (br s, 1 H, α-OH), 13.51 (br s, 1 H, α-OH), 12.22 (br s, 1 H, β-OH), 5.62 (br s, 1 H, OH), 3.56 (s, 2 H), 3.54 (t, J = 6.8 Hz, 2 H), 2. 62 (t, J = 6.8 Hz, 2 H). ^13^C NMR (DMSO-d_6_, 75 MHz): δ = 186.7, 181.8, 157.5, 153.0, 152.8, 132.0, 129.7, 122.2, 110.8, 110.1, 60.9, 35.0, 23.2. HRMS (ESI): m/z [M-H]^−^ calcd for C_13_H_9_Cl_2_O_6_S: 362.9502; found: 362.9503.

2,5-Dihydroxy-3-((2-hydroxyethylthio)methyl)-1,4-naphthoquinone (**22**) obtained from 2,5-dihydroxy-1,4-naphthoquinone (7): yellow solid; yield: 260 mg (93%); mp 124–126 °C. IR (CHCl_3_): 3393, 2930, 1661, 1622, 1603, 1459, 1388, 1332, 1294, 1277, 1241, 1228, 1220, 1206, 1166 cm^−1^. ^1^H NMR (CDCl_3_, 300 MHz): δ = 12.28 (s, 1 H, α-OH), 7.68 (dd, J = 7.5, 1.0 Hz, 1 H), 7.65 (br s, 1 H, β-OH), 7.57 (t, J = 7.5 Hz, 1 H), 7.32 (dd, J = 7.5, 1.0 Hz, 1 H), 3.86 (t, J = 7.8 Hz, 2 H), 3.73 (s, 2 H), 2. 81 (t, J = 7.8 Hz, 2 H). ^13^C NMR (CDCl_3_, 75 MHz): δ = 189.7, 180.5, 161.6, 153.7, 135.4, 129.3, 126.8, 120.6, 119.7, 114.3, 60.8, 35.6, 22.6. HRMS (ESI): m/z [M-H]^−^ calcd for C_13_H_11_O_5_S: 279.0333; found: 279.0336.

3,5-Dihydroxy-2-((2-hydroxyethylthio)methyl)-1,4-naphthoquinone (**23**) obtained from 2,8-dihydroxy-1,4-naphthoquinone (8): yellow solid; yield: 266 mg (95%); mp 132–134 °C. IR (KBr): 3374, 2931, 1624, 1577, 1482, 1460, 1406, 1386, 1369, 1292, 1218, 1209, 1162, 1146, 1066 cm^−1^. ^1^H NMR (CDCl_3_, 300 MHz): δ = 11.07 (s, 1 H, α-OH), 7.72–7.62 (m, 2 H), 7.60 (s, 1 H, β-OH), 7.23 (dd, J = 7.5, 2.0 Hz, 1 H), 3.85 (t, J = 5.6 Hz, 2 H), 3.82 (s, 2 H), 2. 80 (t, J = 5.6 Hz, 2 H). ^13^C NMR (CDCl_3_, 75 MHz): δ = 184.4, 183.0, 161.5, 153.0, 137.9, 132.3, 123.6, 122.3, 120.0, 113.0, 60.8, 35.6, 23.0. HRMS (ESI): m/z [M-H]^−^ calcd for C_13_H_11_O_5_S: 279.0333; found: 279.0334.

2-Hydroxy-3-((2-hydroxyethylthio)methyl)-5,6,7-trimethoxy-1,4-naphthoquinone (**24**) obtained from 2-hydroxy-5,6,7-trimethoxy-1,4-naphthoquinone (9): yellow solid; yield: 322 mg (91%); mp 126–128 °C. IR (KBr): 3413, 2941, 1659, 1574, 1484, 1468, 1454, 1416, 1373, 1328, 1251, 1215, 1200, 1142, 1100, 972 cm^−1^. ^1^H NMR (CDCl_3_, 300 MHz): δ = 7.48 (s, 1 H), 7.40 (br s, 1 H, β-OH), 4.00 (s, 3 H), 3.98 (s, 3 H), 3.93 (s, 3 H), 3.84 (t, J = 5.8 Hz, 2 H), 3.70 (s, 2 H), 2. 81 (t, J = 5.8 Hz, 2 H). ^13^C NMR (CDCl_3_, 75 MHz): δ = 182.6, 180.7, 156.7, 154.7, 151.6, 149.7, 126.3, 122.0, 119.7, 106.2, 61.6, 61.4, 60.7, 56.4, 35.7, 23.1. HRMS (ESI): m/z [M-H]^−^ calcd for C_16_H_17_O_7_S: 353.0700; found: 353.0703.

3,5,8-Trihydroxy-2-((2-hydroxyethylthio)methyl)-6-methoxy-1,4-naphthoquinone (**25**) obtained from 2,5,8-trihydroxy-7-methoxy-1,4-naphthoquinone (**10**): red solid; yield: 300 mg (92%); mp 195–198 °C. IR (KBr): 3530, 1594, 1484, 1455, 1437, 1408, 1385, 1363, 1309, 1195, 1064, 1024 cm^−1^. ^1^H NMR (CDCl_3_, 300 MHz): δ = 13.22 (s, 1 H, α-OH), 12.03 (s, 1 H, α-OH), 7.32 (s, 1 H, β-OH), 6.59 (s, 1 H), 3.99 (s, 3 H, OCH_3_), 3.87 (t, J = 7.8 Hz, 2 H), 3.85 (s, 2 H), 2.80 (t, J = 7.8 Hz, 2 H). ^13^C NMR (CDCl_3_, 75 MHz): δ = 181.6, 177.2, 164.2, 157.1, 155.4, 152.9, 122.8, 110.3, 109.4, 108.8, 60.8, 56.8, 35.6, 23.0. HRMS (ESI): m/z [M-H]^−^ calcd for C_14_H_13_O_7_S: 325.0387; found: 325.0387.

#### 3.1.4. General Procedure for the Synthesis of 2-Chloroethylthio-1,4-naphthoquinones and (2-Chloroethylthio)methyl-1,4-naphthoquinones

To a solution (or suspension) of appropriate 2-hydroxyethylthio-1,4-naphthoquinone **13**–**20** or (2-hydroxyethylthio)methyl-1,4-naphthoquinone **21**–**25** (0.5 mmol) in methylene chloride (50 mL), thionyl chloride (300 mg, 182 μL, 2.5 mmol) was added. The mixture was stirred at room temperature for 4 h until TLC indicated that the reaction was complete. The mixture was evaporated in vacuo and the solid was purified by column chromatography (silica gel, hexane-acetone, 20:1). For details, see Table S1.4

6,7-Di(2-chloroethylthio)-2-ethyl-3,5,8-trihydroxy-1,4-naphthoquinone (**26**) obtained from 6,7-di(2-hydroxyethylthio)-2,5,8-trihydroxy-1,4-naphthoquinone (**13**): dark violet solid; yield: 159 mg (75%); mp 126–128 °C. IR (CHCl_3_): 3418, 2977, 1617, 1600, 1365, 1331, 1280 cm^−1^. ^1^H NMR (CDCl_3_, 300 MHz): δ = 14.07 (s, 1 H, α-OH), 12.49 (s, 1 H, α-OH), 7.35 (s, 1 H, β-OH), 3.73–3.56 (m, 6 H), 3.50–3.43 (m, 2 H), 2.64 (q, J = 7.5 Hz, 2 H), 1.16 (t, J = 7.5 Hz, 3 H). ^13^C NMR (CDCl_3_, 75 MHz): δ = 187.4, 180.5, 158.0, 157.8, 153.3, 143.2, 135.4, 126.9, 109.0, 108.6, 43.3, 43.1, 36.7, 36.5, 16.3, 12.4. HRMS (ESI): m/z [M-H]^−^ calcd for C_16_H_15_Cl_2_O_5_S_2_: 420.9743; found: 420.9744.

6-(2-Chloroethylthio)-2,3,5,8-tetrahydroxy-7-methyl-1,4-naphthoquinone (**27**) obtained from 2,3,5,8-tetrahydroxy-6-(2-hydroxyethylthio)-7-methyl -1,4-naphthoquinone (**14**): purple solid; yield: 136 mg (82%); mp 162–166 °C. IR (CHCl_3_): 3662, 3433, 1693, 1599, 1416, 1376, 1310, 1288 cm^−1^. ^1^H NMR (CDCl_3_, 300 MHz): δ = 12.69 (s, 1 H, α-OH), 12.23 (s, 1 H, α-OH), 6.70 (s, 2 H, β-OH), 3.60 (t, J = 7.5 Hz, 2 H), 3 41 (t, J = 7.5 Hz, 2 H), 2.54 (s, 3 H).

^13^C NMR (DMSO-d_6_, 75 MHz): δ = 183.3, 183.1, 156.5, 154.5, 142.3, 141.2, 141.2, 132.8, 108.0, 106.9, 43.9, 35.7, 14.5. HRMS (ESI): m/z [M-H]^−^ calcd for C_13_H_10_ClO_6_S: 328.9892; found: 328.9896.

Equimolar mixture of 6-(2-chloroethylthio)-2,5,8-trihydroxy3-methyl-1,4-naphthoquinone (**28a**) and 6-(2-chloroethylthio)-3,5,8-trihydroxy-2-methyl-1,4-naphthoquinone (**28b**) obtained from the mixture of 2,5,8-trihydroxy-6-(2-hydroxyethylthio)3-methyl-1,4-naphthoquinone (**15a**) and 3,5,8-trihydroxy-6-(2-hydroxyethylthio)-2-methyl-1,4-naphthoquinone (**15b**)): dark red solid; yield: 134 mg (85%); HRMS (ESI): m/z [M-H]^−^ calcd for C_13_H_10_ClO_5_S: 312.9943; found: 312.9945.

6-(2-chloroethylthio)-2,5,8-trihydroxy-3-methyl-1,4-naphthoquinone (**28a**). ^1^H NMR (CDCl_3_, 700 MHz): δ = 13.64 (s, 1 H, α-OH), 11.61 (s, 1 H, α-OH), 7.45 (s, 1 H, β-OH), 6.88 (s, 1 H), 3.75 (t, J = 7.5 Hz, 2 H), 3.37 (t, J = 7.5 Hz, 2 H), 2.08 (s, 3 H). ^13^C NMR (CDCl_3_, 175 MHz): δ = 188.3, 179.3, 158.4, 155.4, 154.5, 144.3, 120.6, 119.4, 109.2, 107.4, 40.9, 32.7, 8.1.

6-(2-chloroethylthio)-3,5,8-trihydroxy-2-methyl-1,4-naphthoquinone (**28b**). ^1^H NMR (CDCl_3_, 700 MHz): δ = 12.87 (s, 1 H, α-OH), 12.10 (s, 1 H, α-OH), 7.25 (s, 1 H, β-OH), 7.03 (s, 1 H), 3.73 (t, J = 7.5 Hz, 2 H), 3.36 (t, J = 7.5 Hz, 2 H), 2.10 (s, 3 H). ^13^C NMR (CDCl_3_, 175 MHz): δ = 186.9, 181.3, 157.9, 155.9, 153.1, 138.5, 124.5, 122.4, 109.4, 107.9, 41.1, 32.9, 8.3.

6,7-Di(2-chloroethylthio)-2,5,8-trihydroxy-1,4-naphthoquinone (**29**) obtained from 6,7-di(2-hydroxyethylthio)-2,5,8-trihydroxy-1,4-naphthoquinone (**16**)): purple solid; yield: 134 mg (68%); mp 133–136 °C. IR (CHCl_3_): 3518, 3413, 2953, 1659, 1605, 1534, 1442, 1403, 1384, 1343, 1269, 1194, 1106 cm^−1^. ^1^H NMR (CDCl_3_, 300 MHz): δ = 13.84 (s, 1 H, α-OH), 12.52 (s, 1 H, α-OH), 7.41 (s, 1 H, β-OH), 6.43 (s, 1 H), 3.75–3.61 (m, 6 H), 3. 48 (t, J = 7.5 Hz, 2 H). ^13^C NMR (CDCl_3_, 75 MHz): δ = 185.6, 177.2, 160.8, 159.8, 157.0, 137.9, 135.8, 111.0, 109.1, 108.7, 43.5, 43.3, 36.9, 36.5. HRMS (ESI): m/z [M-H]^−^ calcd for C_14_H_11_Cl_2_O_5_S_2_: 392.9430; found: 392.9431.

Equimolar mixture of 7-(2-chloroethylthio)-2-ethyl-5,8-dihydroxy-1,4-naphthoquinone (**30a**) and 6-(2-chloroethylthio)-2-ethyl-5,8-dihydroxy-1,4-naphthoquinone (**30b**) obtained from the mixture of 2-ethyl-5,8-dihydroxy-7-(2-hydroxyethylthio)1,4-naphthoquinone (**17a**) and 2-ethyl-5,8-dihydroxy-6-(2-hydroxyethylthio)-1,4-naphthoquinone (**17b**)): red solid; yield: 134 mg (86%); HRMS (ESI): m/z [M-H]^−^ calcd for C_14_H_12_ClO_4_S: 311.0150; found: 311.0151.

7-(2-Chloroethylthio)-2-ethyl-5,8-dihydroxy-1,4-naphthoquinone (**30a**). ^1^H NMR (CDCl_3_, 300 MHz): δ = 12.73 (s, 1 H, α-OH), 12.62 (s, 1 H, α-OH), 7.07 (s, 1 H), 6.68 (s, 1 H), 3.76 (t, J = 7.5 Hz, 2 H), 3.28 (t, J = 7.5 Hz, 2 H), 2.71 (q, J = 7.5 Hz, 2 H), 1.25 (t, J = 7.5 Hz, 3 H). ^13^C NMR (CDCl_3_, 75 MHz): δ = 179.1, 179.0, 163.8, 163.3, 151.2, 147.3, 129.6, 126.6, 110.8, 109.3, 40.4, 32.4, 22.9, 12.5

6-(2-Chloroethylthio)-2-ethyl-5,8-dihydroxy-1,4-naphthoquinone (**30b**). ^1^H NMR (CDCl_3_, 300 MHz): δ = 13.03 (s, 1 H, α-OH), 12.36 (s, 1 H, α-OH), 7.01 (s, 1 H), 6.66 (s, 1 H), 3.76 (t, J = 7.5 Hz, 2 H), 3.28 (t, J = 7.5 Hz, 2 H), 2.73 (q, J = 7.5 Hz, 2 H), 1.26 (t, J = 7.5 Hz, 3 H). ^13^C NMR (CDCl_3_, 75 MHz): δ = 180.1, 178.5, 164.1, 162.4, 152.0, 149.6, 127.5, 126.2, 110.1, 109.9, 40.4, 32.4, 23.1, 12.5.

Equimolar mixture of 2,6-di(2-chloroethylthio)-3-ethyl-5,8-dihydroxy-1,4-naphthoquinone (**31a**) and 3,6-di(2-chloroethylthio)-2-ethyl-5,8-dihydroxy-1,4-naphthoquinone (**31b**) obtained from the mixture of 3-ethyl-5,8-dihydroxy-2,6-di(2-hydroxyethylthio)1,4-naphthoquinone (**18a**) and 2-ethyl-5,8-dihydroxy-3,6-di(2-hydroxyethylthio)-1,4naphthoquinone (**18b**): dark red solid; yield: 141 mg (69%); HRMS (ESI): m/z [M-H]^−^ calcd for C_16_H_15_Cl_2_O_4_S_2_: 404.9794; found: 404.9792.

2,6-Di(2-chloroethylthio)-3-ethyl-5,8-dihydroxy-1,4-naphthoquinone (**31a**). ^1^H NMR (CDCl_3_, 300 MHz): δ = 13.21 (s, 1 H, α-OH), 13.06 (s, 1 H, α-OH), 6.77 (s, 1 H), 3.76 (t, J = 7.5 Hz, 2 H), 3.65 (t, J = 7.5 Hz, 2 H), 3.45 (t, J = 7.5 Hz, 2 H), 3.31 (t, J = 7.5 Hz, 2 H), 3.06 (q, J = 7.5 Hz, 2 H), 1.17 (t, J = 7.5 Hz, 3 H). ^13^C NMR (CDCl_3_, 75 MHz): δ = 174.8, 173.8, 168.6, 166.2, 153.3, 149.1, 136.8, 125.4, 109.9, 109.6, 43.4, 40.5, 36.0, 32.5, 22.6, 13.5.

3,6-Di(2-chloroethylthio)-2-ethyl-5,8-dihydroxy-1,4-naphthoquinone (**31b**). ^1^H NMR (CDCl_3_, 300 MHz): δ = 13.34 (s, 1 H, α-OH), 13.00 (s, 1 H, α-OH), 6.76 (s, 1 H), 3.76 (t, J = 7.5 Hz, 2 H), 3.65 (t, J = 7.5 Hz, 2 H), 3.51 (t, J = 7.5 Hz, 2 H), 3.31 (t, J = 7.5 Hz, 2 H), 3.03 (q, J = 7.5 Hz, 2 H), 1.16 (t, J = 7.5 Hz, 3 H). ^13^C NMR (CDCl_3_, 75 MHz): δ = 174.5, 173.2, 167.9, 167.7, 150.4, 149.3, 139.6, 124.8, 110.3, 108.9, 43.5, 40.5, 36.0, 32.5, 22.4, 13.3.

2-(2-Chloroethylthio)-5,8-dihydroxy-6,7-dimethyl-1,4-naphthoquinone (**32**) obtained from 5,8-dihydroxy-2-(2-hydroxyethylthio)-6,7-dimethyl-1,4-naphthoquinone (**19**): purple solid; yield: 120 mg (77%); mp 125–127 °C. IR (CHCl_3_): 2927, 2856, 1733, 1715, 1601, 1555, 1457, 1391, 1300, 1267 cm^−1^. ^1^H NMR (CDCl_3_, 300 MHz): δ = 13.09 (s, 1 H, α-OH), 13.07 (s, 1 H, α-OH), 6.79 (s, 1 H), 3.75 (t, J = 7.5 Hz, 2 H), 3.31 (t, J = 7.5 Hz, 2 H), 2.54 (s, 3 H). ^13^C NMR (CDCl_3_, 75 MHz): δ = 172.6, 171.5, 171.1, 170.3, 147.8, 142.5, 140.3, 124.8, 109.7, 108.7, 40.8, 32.6, 12.7, 12.5. HRMS (ESI): m/z [M-H]^−^ calcd for C_14_H_12_ClO_4_S: 311.0150; found: 311.0152.

2,3-Di(2-chloroethylthio)-5,8-dihydroxy-6,7-dimethyl-1,4-naphthoquinone (**33**) obtained from 5,8-dihydroxy-2,3-di(2-hydroxyethylthio)-6,7-dimethyl-1,4-naphthoquinone (**20**): purple solid; yield: 132 mg (65%); mp 132–136 °C. IR (CHCl_3_): 2928, 2856, 1600, 1445, 1392, 1379, 1297, 1270 cm^−1^. ^1^H NMR (CDCl_3_, 300 MHz): δ = 13.51 (s, 2 H, 2 α-OH), 3.72 (t, J = 7.4 Hz, 4 H), 3.59 (t, J = 7.4 Hz, 4 H), 2.26 (s, 6 H, Me). ^13^C NMR (CDCl_3_, 75 MHz): δ = 171.9, 170.1, 143.0, 141.3, 109.4, 43.5, 36.6, 12.5. HRMS (ESI): m/z [M-H]^−^ calcd for C_16_H_15_Cl_2_O_4_S_2_: 406.3246; found: 406.3250.

3-((2-Chloroethylthio)methyl)-2-hydroxy-5,6,7-trimethoxy-1,4-naphthoquinone (**34**) obtained from 2-hydroxy-3-((2-hydroxyethylthio)methyl)-5,6,7-trimethoxy-1,4-naphthoquinone (**24**)): yellow solid; yield: 158 mg (85%); mp 129–132 °C. IR (CHCl_3_): 3410, 3001, 1704, 1655, 1578, 1483, 1466, 1418, 1374, 1334, 1294, 1143,1102 cm^−1^. ^1^H NMR (CDCl_3_, 300 MHz): δ = 7.48 (s, 1 H), 7.28 (br s, 1 H, β-OH), 4.00 (s, 3 H), 3.98 (s, 3 H), 3.93 (s, 3 H), 3.72 (t, J = 7.8 Hz, 2 H), 3.69 (s, 2 H), 2. 92 (t, J = 7.8 Hz, 2 H). ^13^C NMR (CDCl_3_, 75 MHz): δ = 182.3, 180.7, 156.7, 154.8, 151.5, 149.8, 126.2, 121.8, 119.7, 106.2, 61.7, 61.4, 56.5, 42.9, 34.4, 23.5. HRMS (ESI): m/z [M-H]^−^ calcd for C_16_H_17_ClO_6_S: 371.0361; found: 371.0364.

3-((2-Chloroethylthio)methyl)-2,5-dihydroxy-1,4-naphthoquinone (**35**) obtained from 2,5-dihydroxy-3-((2-hydroxyethylthio)methyl)-1,4-naphthoquinone (**22**): yellow solid; yield: 114 mg (76%); mp 116–118 °C. IR (CHCl_3_): 3391, 3010, 1660, 1623, 1459, 1415, 1387, 1332, 1294, 1277, 1191, 1167,1070 cm^−1^. ^1^H NMR (CDCl_3_, 300 MHz): δ = 12.27 (s, 1 H, α-OH), 7.68 (dd, J = 7.5, 1.0 Hz, 1 H), 7.65 (br s, 1 H, β-OH), 7.57 (t, J = 7.5 Hz, 1 H), 7.32 (dd, J = 7.5, 1.0 Hz, 1 H), 3.73 (t, J = 7.8 Hz, 2 H), 3.71 (s, 2 H), 2. 93 (t, J = 7.8 Hz, 2 H). ^13^C NMR (CDCl_3_, 75 MHz): δ = 189.6, 180.3, 161.6, 153.6, 135.4, 129.3, 126.7, 120.6, 119.7, 114.2, 42.8, 34.4, 22.9. HRMS (ESI): m/z [M-H]^−^ calcd for C_13_H_10_ClO_4_S: 296.9996; found: 296.9994.

2-((2-Chloroethylthio)methyl)-3,5-dihydroxy-1,4-naphthoquinone (**36**) obtained from 3,5-dihydroxy-2-((2-hydroxyethylthio)methyl)-1,4-naphthoquinone (**23**): yellow solid; yield: 117 mg (78%); mp 119–121 °C. IR (CHCl_3_): 3412, 3003, 1653, 1630, 1577, 1461, 1417, 1367, 1318, 1277, 1170 cm^−1^. ^1^H NMR (CDCl_3_, 500 MHz): δ = 11.05 (s, 1 H, α-OH), 7.70–7.64 (m, 2 H), 7.52 (s, 1 H, β-OH), 7.23 (dd, J = 8.0, 1.5 Hz, 1 H), 3.72 (t, J = 7.6 Hz, 2 H), 3.71 (s, 2 H), 2. 93 (t, J = 7.6 Hz, 2 H). ^13^C NMR (CDCl_3_, 125 MHz): δ = 184.3, 182.8, 161.5, 152.8, 138.0, 132.3, 123.6, 122.1, 120.0, 113.0, 42.8, 34.4, 23.4. HRMS (ESI): m/z [M-H]^−^ calcd for C_13_H_10_ClO_4_S: 296.9994; found: 296.9994.

6,7-Dichloro-2-((2-chloroethylthio)methyl)-3,5,8-trihydroxy-1,4-naphthoquinone (**37**) obtained from 6,7-dichloro-2,5,8-trihydroxy-3-((2-hydroxyethylthio)methyl)1,4-naphthoquinone (**21**): red solid; yield: 139 mg (72%); mp 143–147 °C. IR (CHCl_3_): 3410, 3010, 1657, 1630, 1610, 1553, 1432, 1402, 1323, 1271, 1226, 1212, 1204, 1180, 1113 cm^−1^.

^1^H NMR (CDCl_3_, 500 MHz): δ = 13.34 (s, 1 H, α-OH), 12.01 (s, 1 H, α-OH), 7.55 (s, 1 H, β-OH), 3.74 (s, 2 H), 3.72 (t, J = 7.8 Hz, 2 H), 2. 94 (t, J = 7.8 Hz, 2 H). ^13^C NMR (CDCl_3_, 125 MHz): δ = 186.3, 180.7, 154.7 (2C), 153.7, 135.7, 131.7, 122.5, 108.9, 108.8, 42.7, 34.6, 23.0. HRMS (ESI): m/z [M-H]^−^ calcd for C_13_H_8_Cl_3_O_5_S: 380.9163; found: 380.9165.

2-((2-Chloroethylthio)methyl)-3,5,8-trihydroxy-6-methoxy-1,4-naphthoquinone (**38**) obtained from 3,5,8-trihydroxy-2-((2-hydroxyethylthio)methyl)-6-methoxy-1,4-naphthoquinone (**25**): red solid; yield: 128 mg (74%); mp 176–179 °C. IR (CHCl_3_): 3523, 3411, 2942, 1602, 1478, 1436, 1413, 1396, 1354, 1323, 1304, 1274, 1217, 1210, 1196, 1178 cm^−1^. ^1^H NMR (CDCl_3_, 300 MHz): δ = 13.20 (s, 1 H, α-OH), 12.02 (s, 1 H, α-OH), 7.30 (s, 1 H, β-OH), 6.59 (s, 1 H), 3.98 (s, 3 H, OCH_3_), 3.75 (s, 2 H), 3.73 (t, J = 7.8 Hz, 2 H), 2. 93 (t, J = 7.8 Hz, 2 H). ^13^C NMR (CDCl_3_, 75 MHz): δ = 181.5, 177.1, 164.0, 157.0, 155.4, 152.9, 122.8, 110.3, 109.4, 108.8, 56.7, 42.8, 34.4, 23.1. HRMS (ESI): m/z [M-H]^−^ calcd for C_14_H_12_ClO_6_S: 343.0048; found: 343.0051.

### 3.2. Biology

#### 3.2.1. Reagents and Antibodies

The following reagents were used: RNase (Carl Roth, Karlsruhe, Germany); PhosSTOP™ *EASY*packs phosphatase inhibitors cocktail and cOmplete™ *EASY*packs protease inhibitors cocktail (Roche, Mannheim, Germany); CM-H_2_DCFDA (Molecular Probes, Invitrogen, Eugene, OR, USA); N-acetyl cysteine and GSK-690693 (Akt inhibitor) (MedChemExpress, Monmouth Junction, South Brunswick, NJ, USA); Anisomycin (NeoCorp, Weilheim, Germany); carboplatin, cisplatin, and docetaxel (Pharmacy of the University Hospital Hamburg-Eppendorf, Hamburg, Germany); propidium iodide (PI), CCCP (2-[2-(3-chlorophenyl)hydrazinylyidene]propanedinitrile), and MTT (3-(4,5-dimethylthiazol-2-yl)-2,5-diphenyltetrazolium bromide) (Sigma, Taufkirchen, Germany); enzalutamide (Hycultec GmbH, Beutelsbach, Germany); PD98059 (MEK inhibitor) (Merck Chemicals GmbH, Darmstadt, Germany); SCH772984 (ERK1/2 inhibitors) (Adooq Bioscience, Irvine, CA, USA); olaparib (PARP inhibitor), SP600125 (JNK1/2 inhibitor), and SB203580 (p38 inhibitor) (LC Laboratories, Woburn, MA, USA); annexin-V-FITC (BD Bioscience, San Jose, CA, USA). The primary and secondary antibodies used are listed in Table 3.

#### 3.2.2. Cell Lines and Culture Conditions

Human prostate cancer cell lines PC-3, DU145, 22Rv1, and LNCaP, as well as human prostate non-cancer cells RWPE-1 and PNT2, and non-cancer human fibroblast MRC-9 cells were purchased from ATCC (Manassas, VA, USA). Human prostate cancer cell line VCaP was purchased from ECACC (Salisbury, UK). Human embryonic kidney HEK293T cells and human umbilical vascular endothelial HUVEC cells were donated by Prof. Dr. Sonja Loges (University Medical Center Hamburg-Eppendorf, Hamburg, Germany). Cells were authenticated by a commercial service (Multiplexion, Heidelberg, Germany) and cultured according to the manufacturer’s protocols. For details, please see Supplementary Materials.

#### 3.2.3. In Vitro Cytotoxicity Assay (MTT Test)

A total of 6000 cells/well were seeded in 96-well plates, incubated overnight, and treated with the drugs in 100 μL/well of the corresponding fresh culture media for 48 h, as previously described [58]. For combinational assays, the cells were pre-treated with the corresponding inhibitor for the indicated time and then co-treated with the tested compounds for 48 h. Then, 10 μL/well of MTT ((3-(4,5-dimethylthiazol-2-yl)-2,5-diphenyltetrazolium bromide) solution was added and the cell viability was measured as previously reported [58].

#### 3.2.4. Drug Combinational Studies

The synergistic or antagonistic effects of the investigated drugs in combination with approved chemotherapeutic drugs or specific inhibitors were performed using the Chou–Talalay method [59,60] and as previously described [61]. Cells were pre-treated with the corresponding inhibitor prior to the drug application or co-treated with the drugs and inhibitor for the indicated time at a constant molar ratio. The molar ratios of the individual drugs used for combinational studies are shown in Supplementary Information (Table S2). The in vitro cytotoxic activity of the individual compounds and their combinations was evaluated using the MTT assay. The generated data were analyzed with CompuSyn v.1.0 (ComboSyn Inc., Paramus, NJ, USA) software and a combinational index (CI) was calculated. CI > 1.2 was assumed as antagonism; CI = 0.85~1.2 corresponds to the additive effect; CI < 0.85 refers to synergism. The analysis was performed according to previously published recommendations [59,60].

#### 3.2.5. DNA Fragmentation and Cell Cycle Analysis

The experiment was performed as previously reported [58]. In brief, 0.2 × 10^6^ cells/well were seeded in 12-well plates at 1 mL/well, incubated overnight, and treated with the tested compounds in fresh culture media (1 mL/well) for 48 h. Then, the cells were harvested using trypsinization, fixed with 70% EtOH overnight, stained with propidium iodide (PI), and analyzed using the flow cytometry technique. For the analysis, the FACS Calibur (BD Bioscience, San Jose, CA, USA) instrument and BD Bioscience Cell Quest Pro v.5.2.1 software (BD Bioscience) were used.

#### 3.2.6. Detection of Apoptotic Cells by Annexin-V-FITC/PI Double Staining

The experiment was performed as previously reported with slight modifications [16]. In brief, 0.2 × 10^6^ cells/well were seeded in 6-well plates and incubated overnight. The medium was exchanged with a fresh culture medium (1 mL/well) and the cells were pre-treated with z-VAD(OMe)-fmk for 1 h, followed by treatment with the investigated drugs for 48 h. The cells were then harvested by trypsinization as described for the DNA fragmentation and cell cycle analysis (see Section 3.2.5), double-stained using annexin-V-FITC and PI, and analyzed using the flow cytometry technique with either FACS Canto or FACS Calibur machines (BD Bioscience, San Jose, CA, USA) and BD Bioscience Cell Quest Pro v.5.2.1 software (BD Bioscience).

#### 3.2.7. Western Blotting

Western blotting was performed as previously reported [34,62]. The cells were seeded in ø 6 cm Petri dishes (1 × 10^6^ cells/dish in 5 mL/dish of culture media), incubated overnight, and treated with drugs at 5 mL/dish of a fresh culture medium for either 2 h or 48 h. The cells were harvested and lysed in lysis buffer. Then, the protein concentration was determined by the Bradford assay, and 20 μg total protein of each sample was loaded on the gradient polyacrylamide ready-made gel (Bio-Rad, Hercules, CA, USA). The proteins were separated, transferred onto a PVDF membrane, and detected using the corresponding primary and secondary antibodies.

#### 3.2.8. Evaluation of Intracellular ROS

The levels of intracellular ROS were evaluated using the reactive oxygen species-sensitive CM-H_2_DCFDA reagent and the flow cytometry technique. 22Rv1 cells were plated in 12-well plates and consequently incubated with CM-H_2_DCFDA solution in PBS, and then treated with the drug solution in PBS for the indicated time. Next, the cells were harvested and immediately analyzed according to the manufacturer’s protocols as previously described [63].

#### 3.2.9. Mitochondrial Membrane Potential Analysis

The loss of Δψ_m_ (mitochondrial membrane potential) was accessed using Δψ-sensitive JC-1 dye (5,5′,6,6′-tetrachloro-1,1′,3,3′-tetraethyl-imidacarbocyanine iodide) staining and the flow cytometry technique. The cells were plated in 12-well plates and treated with a solution of the investigated drugs in PBS for 2 h. Then, the cells were harvested, stained with JC-1, and immediately analyzed as previously described [12].

#### 3.2.10. Statistical Analysis

All the experiments were performed in triplicate and repeated at least three times. Statistical analyses were performed using GraphPad Prism software v. 5.01 (GraphPad Prism Software Inc., La Jolla, CA, USA) using two types of analyses: the one-way analysis of variance (ANOVA) test, followed by a post hoc Dunnett’s test for multiple group comparisons; or an unpaired Student’s t-test for the comparison of two groups. Data are presented as mean ± SEM (standard error of the mean). The differences were considered statistically significant if *p* < 0.05 for either test used.

## 4. Conclusions

In conclusion, we synthesized novel cytotoxic (2-chloroethylthio)-1,4-naphthoquinone hybrid molecules, revealing promising activity in human CRPC cell lines at nanomolar concentrations. The mechanism of anticancer activity was suggested to be a classical caspase-dependent apoptosis putatively exerted via cytotoxic ROS induction, mitochondria targeting, and DNA damage. Synergistic and additive effects were observed with the PARP inhibitor olaparib and the AR inhibitor enzalutamide, respectively. The latter was presumably mediated by the down-regulation of AR-V7 expression as well as inhibition of AR-FL/AR-V7-mediated signaling. Additionally, the synthesized compounds could sensitize the cells to the combination of enzalutamide and the AKT inhibitor GSK-690693. Antagonistic effects in combination with some taxane and platinum chemotherapies were found to be at least partly mediated by activation of p38, JNK1/2, ERK1/2, MEK1/2, and AKT kinases. Inhibition of these kinases resulted in amplified efficacy of 30 and 32, suggesting the evaluation of combinational strategies in further pre-clinical and clinical trials. In fact, currently, different ARTAs are evaluated in combination with AKT inhibitors in clinical trials in hormone-sensitive and castration-resistant prostate cancer. Here, the addition of 30 and 32 may be a promising strategy.

## Figures and Tables

**Table 1:** Cytotoxicity of the synthesized compounds in cancer (22Rv1) versus non-cancer (PNT2) prostate cells. Selectivity index values (ratio of IC_50_ in PNT2 cells to IC_50_ in 22Rv1 cells). “#” indicates that IC_50_ values toward the tested cell lines were >100 μM. The values of IC_50_ correspond to those presented in Table 2. All IC_50_ values were evaluated after 48 h of treatment using the MTT test.

Compound	IC_50_(22Rv1), [μM]	IC_50_(PNT2), [μM]	Selectivity Index [IC_50_(PNT2)/IC_50_(22Rv1)]
**11**	>100	>100	n/a
**12**	89.5 ± 24.9	>100	n/a
**26**	41.2 ± 1.18	>100	n/a
**27**	>100	>100	n/a
**28**	93.35 ± 28.6	>100	n/a
**29**	32.06 ± 1.66	6.44 ± 1.24	0.2
**30**	0.36 ± 0.09	0.49 ± 0.09	1.36
**31**	0.51 ± 0.18	0.38 ± 0.03	0.75
**32**	0.14 ± 0.09	0.77 ± 0.07	5.5
**33**	0.75 ± 0.16	0.85 ± 0.04	1.13
**34**	>100	>100	n/a
**35**	>100	>100	n/a
**36**	>100	>100	n/a
**37**	48.55 ± 12.2	28.15 ± 1.04	0.58
**38**	>100	>100	n/a

**Table 2:** IC_50_ values of **30**, **31**, **32**, and **33** in different cell lines. The cells were treated with the investigated compounds for 48 h. IC_50_ values were evaluated using the MTT assay.

Cell Line	IC_50_ [μM], 48 h			
	30	31	32	33
**Cancer Cell Lines**				
22Rv1	0.36 ± 0.09	0.51 ± 0.18	0.14 ± 0.09	0.75 ± 0.16
DU145	0.51 ± 0.13	0.79 ± 0.21	0.71 ± 0.2	0.63 ± 0.05
PC-3	0.56 ± 0.09	0.38 ± 0.1	0.58 ± 0.11	2.27 ± 0.19
VCaP	0.93 ± 0.04	0.79 ± 0.18	0.92 ± 0.16	1.57 ± 0.22
LNCaP	0.63 ± 0.19	0.85 ± 0.07	0.47 ± 0.15	0.85 ± 0.13
**Non-Cancer Cell Lines**				
HEK293	0.24 ± 0.03	0.15 ± 0.01	0.21 ± 0.06	0.58 ± 0.14
MRC9	1.62 ± 0.34	1.41 ± 0.52	1.11 ± 0.23	2.85 ± 0.39
PNT2	0.49 ± 0.09	0.38 ± 0.03	0.77 ± 0.07	0.85 ± 0.04

**Table 3:** List of antibodies used.

Antibodies	Clonality	Source	Cat. No.	Dilution	Manufacturer
anti-ERK1/2	mAb	mouse	#9107	1:2000	Cell Signaling
anti-JNK1/2	mAb	rabbit	#9258	1:1000	Cell Signaling
anti-p38	mAb	rabbit	#9212	1:1000	Cell Signaling
anti-phospho-ERK1/2	mAb	rabbit	#4377	1:1000	Cell Signaling
anti-phospho-JNK1/2	mAb	rabbit	#4668	1:1000	Cell Signaling
anti-phospho-p38	mAb	rabbit	#4511	1:1000	Cell Signaling
anti-phospho-Akt	mAb	rabbit	#4058	1:1000	Cell Signaling
anti-Akt	pAb	rabbit	#9272	1:1000	Cell Signaling
anti-phospho-MEK1/2	mAb	rabbit	#2338	1:1000	Cell Signaling
anti-MEK1/2	pAb	rabbit	#9122	1:1000	Cell Signaling
anti-β-Actin-HRP	pAb	goat	sc-1616	1:10,000	Santa Cruz
anti-AR	pAb	rabbit	sc-816	1:200	Santa Cruz
anti-AR-V7	mAb	rabbit	198394	1:1000	abcam
anti-Bax	mAb	rabbit	#5023	1:1000	Cell Signaling
anti-Bcl-2	pAb	rabbit	#2876	1:1000	Cell Signaling
anti-cleaved Caspase-3	mAb	rabbit	#9664	1:1000	Cell Signaling
anti-PARP	pAb	rabbit	#9542	1:1000	Cell Signaling
anti-Survivin	pAb	rabbit	NB500-201	1:1000	Novus
anti-mouse IgG-HRP		sheep	NXA931	1:10,000	GE Healthcare
anti-rabbit IgG-HRP		goat	#7074	1:5000	Cell Signaling

## Data Availability

Data are contained within the article or Supplementary Materials.

## Supplementary Materials

The following are available online at https://www.mdpi.com/article/10.3390/ph14100949/s1. Figure S1. Effects of **30** and **32** on cell viability in combination with platinum or taxane agents; Table S1.1. Starting compounds used for the synthesis; Table S1.2. Synthesized compounds; Table S1.3. Synthesized compounds; Table S1.4. Synthesized compounds; cell culture conditions; Table S2. Molar ratios of the individual drugs used for drug combinational studies.

## Abbreviations

ψm	mitochondrial membrane potential
ADC	antibody–drug conjugates
AR-V7	androgen receptor splice variant V7
AR-FL	androgen receptor-full length
AR	androgen receptor
ARTA	androgen receptor targeting therapy
CCCP	carbonyl cyanide *m*-chlorophenyl hydrazine
CI	combinational index
CRPC	castration-resistant prostate cancer
DMSO	dimethylsulfoxide
dsDNA	double-strand DNA
ssDNA	single-strand DNA
FACS	fluorescence-activated cell sorting
HDAC	histone deacetylase
HRR	homologous recombination repair mechanisms
MAPK	mitogen-activated protein kinase
MAPKi	MAP kinases inhibitor
mCRPC	metastatic castration-resistant prostate cancer
NaC	N-acetyl-L-cysteine
PCa	prostate cancer
PI	propidium iodide
PSA	prostate-specific antigen
ROS	reactive oxygen species
SAR	structure–activity relationships
SAPK	stress-activated protein kinase
zVAD	z-VAD(OMe)-fmk (pan-caspase inhibitor).
