# Supplementary material for: Activity of New Synthetic (2-Chloroethylthio)-1,4-naphthoquinones in Prostate Cancer Cells
Source: Pharmaceuticals (Basel). 2021 Sep 22;14(10):949. doi: 10.3390/ph14100949 (PMC8540265; doi:10.3390/ph14100949)
Supplement: Supplementary file 1 [file pharmaceuticals-14-00949-s001.zip › pharmaceuticals-1321029-supplementary.pdf]

Supplementary information

Activity of New Synthetic (2-Chloroethylthio)-1,4-Naphthoquinones in Prostate Cancer Cells

Sergey A. Dyshlovoy[1,2,3,#,*], Dmitry N. Pelageev[3,4,#], Lea S. Jakob[1], Ksenia L. Borisova[4], Jessica Hauschild[1], Tobias Busenbender[1], Moritz Kaune[1], Ekaterina A. Khmelevskaya[3,4], Markus Graefen[2], Carsten Bokemeyer[1], Victor Ph. Anufriev[4], Gunhild von Amsberg[1,2]

[1] Laboratory of Experimental Oncology, Department of Oncology, Hematology and Bone Marrow Transplantation with Section Pneumology, Hubertus Wald-Tumorzentrum, University Medical Center Hamburg-Eppendorf, Martinistrasse 52, 20251 Hamburg, Germany; s.dyshlovoy@uke.de (S.A.D.), lea.jakob@stud.uke.uni-hamburg.de (L.S.J.), j.hauschild@uke.de (J.H.), tobias.busenbender@stud.uke.uni-hamburg.de (T.B.), moritz.kaune@stud.uke.uni-hamburg.de (M.K.), c.bokemeyer@uke.de (C.B.), g.von-amsberg@uke.de (G.v.A.)
[2] Martini-Klinik, Prostate Cancer Center, University Hospital Hamburg-Eppendorf, Martinistrasse 52, 20251 Hamburg, Germany; graefen@martini-klinik.de (M.G.)
[3] School of Natural Sciences, Far Eastern Federal University, FEFU Campus, Ajax Bay 10, Russky Island, 690922 Vladivostok, Russian Federation
[4] G.B. Elyakov Pacific Institute of Bioorganic Chemistry, Far-East Branch, Russian Academy of Sciences, 690022 Vladivostok, Russian Federation; pelageev@mail.ru (D.N.P.), borisovaksenia@mail.ru (K.L.B.), khea-96@mail.ru (E.A.Kh.), anufriev@piboc.dvo.ru (V.Ph.A.)

[#] These authors contributed equally
* Correspondence: dyshlovoy@gmail.com; s.dyshlovoy@uke.de; Tel.: +49 40 7410 51950.

CHEMISTRY 1
Table S1.1. Starting compounds used for the synthesis 1
Table S1.2. Synthesized compounds. 3
Table S1.3. Synthesized compounds. 6
Table S1.4. Synthesized compounds. 7
BIOLOGY 13
Cell culture conditions 13
Figure S1. Effects of 30 and 32 on cell viability in combination with platinum or taxane agents 13
Table S2. Molar ratios of the individual drugs used for drug combinational studies. 14

CHEMISTRY

Table S1.1. Starting compounds used for the synthesis

No	Formula	Spectral Information, References, Notes

**1**	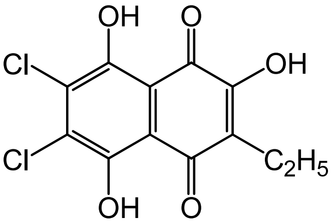	**6,7-dichloro-2-ethyl-3,5,8-trihydroxy-1,4-naphthoquinone**<br>was synthesized as described in Anufriev, V.Ph.; Polonik, S.G.; Pokhilo, N.D.; Balanyova, N.N., Chemistry of naphthazarin derivatives. 11. Trisubstituted hydroquinone derivatives in the preparative synthesis of naphthazarins. *Russ. Chem. Bull. (Translation of Izvestiya Akademii Nauk, Seriya Khimicheskaya)* **2003**, 52, 2247-2250.
**2**	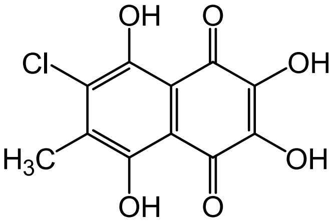	**6-chloro-2,3,5,8-tetrahydroxy-7-methyl-1,4-naphthoquinone**<br>was synthesized from methylchloromaleic anhydride and 1-hydroxy 2,3,4-trimethoxybenzene in a manner analogous to 6,7-dichloro-2-ethyl-3,5,8-trihydroxy-1,4-naphthoquinone (**1**) described by Anufriev, V.Ph.; Polonik, S.G.; Pokhilo, N.D.; Balanyova, N.N., Chemistry of naphthazarin derivatives. 11. Trisubstituted hydroquinone derivatives in the preparative synthesis of naphthazarins. *Russ. Chem. Bull. (Translation of Izvestiya Akademii Nauk, Seriya Khimicheskaya)* **2003**, 52, 2247-2250.
**3**	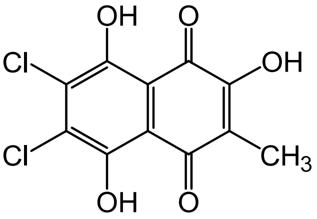	**6,7-dichloro-2,5,8-trihydroxy-3-methyl-1,4-naphthoquinone**<br>was synthesized from dichloromaleic anhydride and 1,2,4-trimethoxy-3-methylbenzene in a manner analogous to 6,7-dichloro-2-ethyl-3,5,8-trihydroxy-1,4-naphthoquinone (**1**) described by Anufriev, V.Ph.; Polonik, S.G.; Pokhilo, N.D.; Balanyova, N.N., Chemistry of naphthazarin derivatives. 11. Trisubstituted hydroquinone derivatives in the preparative synthesis of naphthazarins. *Russ. Chem. Bull. (Translation of Izvestiya Akademii Nauk, Seriya Khimicheskaya)* **2003**, 52, 2247-2250.
**4**	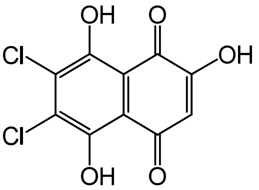	**6,7-dichloro-2,5,8-trihydroxy-1,4-naphthoquinone**<br>was synthesized as described in Anufriev, V.Ph.; Novikov, V.L.; Maximov O.B., Elyakov G.B., Levitsky D.O., Lebedev A.V., Sadretdinov, S.M.; Shvilkin, A.V.; Afonskaya, N.I.; Ruda, M.Ya.; Cherpachenko, N.M., Synthesis of some hydroxynaphthazarins and their cardioprotective effects under ischemia-reperfusion *in vivo. Bioorg. Med. Chem. Lett.* **1998**, 8, 587-592.
**5**	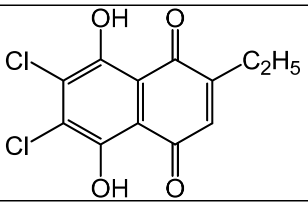	**6,7-dichloro-2-ethyl-5,8-dihydroxy-1,4-naphthoquinone**<br>was synthesized as described in Yakubovskaya, A.Ya.; Pokhilo, N.D.; Anufriev, V.F.; Anisimov, M.M., Synthesis and antimicrobial and antifungal activities of compounds of the naphthazarin series. *Pharmaceutical Chemistry Journal* **2009**, *43*, 396-398.
**6**	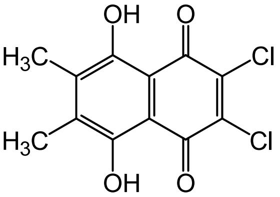	**2,3-dichloro-5,8-dihydroxy-6,7-dimethyl-1,4-naphthoquinone**<br>was synthesized as described in Anufriev, V.Ph.; Polonik, S.G.; Pokhilo, N.D.; Balanyova, N.N., Chemistry of naphthazarin derivatives. 11. Trisubstituted hydroquinone derivatives in the preparative synthesis of naphthazarins. *Russ. Chem. Bull. (Translation of Izvestiya Akademii Nauk, Seriya Khimicheskaya)* **2003**, 52, 2247-2250.
**7**	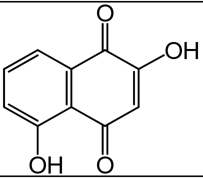	**2,5-dihydroxy-1,4-naphthoquinone**<br>was synthesized as described in Fieser, L.F.; Dunn, J.T., The addition of dienes to halogenated and hydroxylated naphthoquinones. *J. Am. Chem. Soc.* **1937**, 59, 1016-1021.
**8**	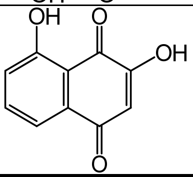	**2,8-dihydroxy-1,4-naphthoquinone**<br>was synthesized as described in Fieser, L.F.; Dunn, J.T., The addition of dienes to halogenated and hydroxylated naphthoquinones. *J. Am. Chem. Soc.* **1937**, 59, 1016-1021.

**9**	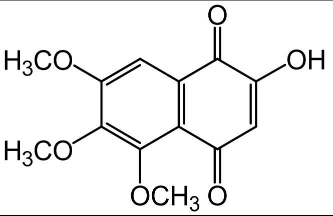	**2-hydroxy-5,6,7-trimethoxy-1,4-naphthoquinone**<br>was synthesized from 2-(3,4,5-trimethoxyphenyl)acetic acid as described in Kun, L.; Kun, Ya.; Lifang Zh.; Yuanyuan, L.; Qi W.; Ruili, L.; Dian, H., Anti-acute myeloid leukemia activity of 2-chloro-3-alkyl-1,4-naphthoquinone derivatives through inducing mtDNA damage and GSH depletion. *Bioorganic and Medicinal Chemistry* **2018**, 26, 4191–4200.
**10**	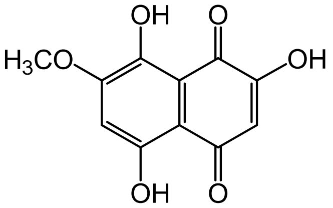	**2,5,8-trihydroxy-7-methoxy-1,4-naphthoquinone**<br>was synthesized as described in Glazunov, V.P.; Tchizhova, A.Ya.; Shestak, O.P.; Sopel'nyak, G.I.; Anufriev, V.Ph., Chemistry of naphthazarin derivatives. 8. Determination of structures of substituted 2-hydroxy-6(7)-methoxynaphthazarins and 7(8)-hydroxypyranonaphthazarins by IR spectroscopy. *Russ. Chem. Bull.* (Translation of *Izvestiya Akademii Nauk, Seriya Khimicheskaya*) 2001, *50*, 95-100.
**11**	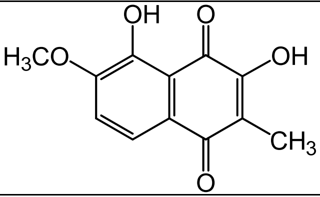	**3,5-dihydroxy-6-methoxy-2-methyl-1,4-naphthoquinone (ancistroquinone B)**<br>was synthesized as described in Khmelevskaya, E.A.; Pelageev, D.N., A convenient synthetic approach to dioncoquinone B and related compounds. Tetrahedron Lett. **2019**, 60, 1022-1024.
**12**	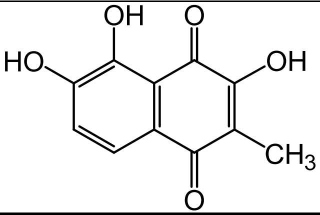	**3,5,6-trihydroxy-2-methyl-1,4-naphthoquinone (dioncoquinone B)**<br>was synthesized as described in Khmelevskaya, E.A.; Pelageev, D.N., A convenient synthetic approach to dioncoquinone B and related compounds. Tetrahedron Lett. **2019**, 60, 1022-1024.

Table S1.2. Synthesized compounds.

No	Formula	Spectral Information, References, Notes
**13**	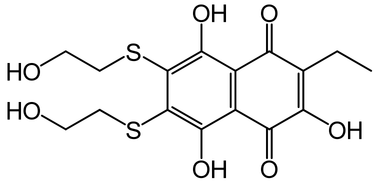	**2-Ethyl-3,5,8-trihydroxy-6,7-di(2-hydroxyethylthio)-1,4-naphthoquinone (13)** (obtained from 6,7-dichloro-2-ethyl-3,5,8-trihydroxy-1,4-naphthoquinone (**1**)):<br>dark violet solid;<br>yield: 564 mg (73%);<br>mp 142–146 °C.<br>**IR (CHCl$_3$) ν_{max}:** 3419, 2937, 2878, 1601, 1382, 1365, 1330, 1279 cm^{-1}.<br>**^{1}H NMR (CDCl$_3$, 300 MHz):** δ = 14.05 (s, 1 H, α-OH), 12.49 (s, 1 H, α-OH), 7.49 (br s, 1 H, β-OH), 3.71 (t, *J* = 5.0 Hz, 2 H), 3.67 (t, *J* = 5.0 Hz, 2 H),), 3.47 (t, *J* = 5.0 Hz, 2 H), 3.34 (t, *J* = 5.0 Hz, 2 H), 2.63 (q, *J* = 7.3 Hz, 2 H), 1.15 (t, *J* = 7.3 Hz, 3 H).<br>**^{13}C NMR (CDCl$_3$, 75 MHz):** δ **=** 187.9, 181.2, 158.0, 157.9, 153.5, 143.8, 136.3, 127.0, 109.2, 108.8, 61.1, 60.6, 39.0, 38.7, 16.4, 12.5.<br>**HRMS (ESI):** *m/z* [M-H]$^-$ calcd for $C_{16}H_{17}O_7S_2$: 385.0421; found: 385.0424.
**14**	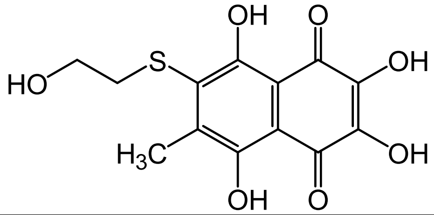	**2,3,5,8-tetrahydroxy-6-(2-hydroxyethylthio)-7-methyl - 1,4-naphthoquinone (14)** (obtained from 6-chloro-2,3,5,8-tetrahydroxy-7-methyl-1,4-naphthoquinone (**2**)):<br>dark red solid;<br>yield: 393 mg (63%);<br>mp 195-200°C.<br>**IR (KBr):** 3480, 3402, 1677, 1658, 1599, 1451, 1406,

		1303, 1279, 1218 cm^{-1}.<br>**^{1}H NMR (acetone-d_6, 300 MHz):** δ = 13.08 (s, 1 H, α-OH), 12.68 (s, 1 H, α-OH), 9.03 (s, 2 H, β-OH), 3.86 (br s, 1 H, OH), 3.65 (t, *J* = 6.5 Hz, 2 H), 3.21 (t, *J* = 6.5 Hz, 2 H), 2.49 (s, 3 H, CH_3).<br>**^{13}C NMR (DMSO-d_6, 75 MHz):** δ = 183.8, 183.4, 157.2, 155.0, 142.2, 141.7, 141.6, 135.1, 108.1, 107.1, 61.0, 36.8, 15.0.<br>**HRMS (ESI):** *m/z* [M-H]$^-$ calcd for $C_{13}H_{11}O_7S$: 311.0231; found: 311.0232.
**15a**<br>**15b**	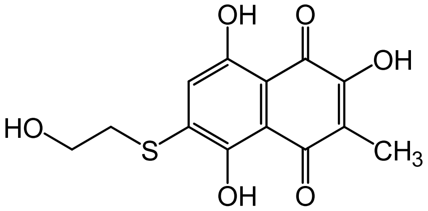<br>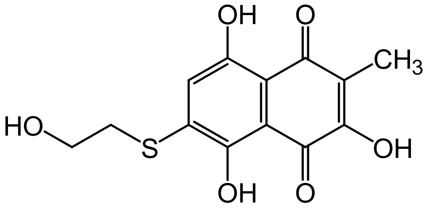	**mixture of 2,5,8-trihydroxy-6-(2-hydroxyethylthio)-3-methyl-1,4-naphthoquinone (15a) and 3,5,8-trihydroxy-6-(2-hydroxyethylthio)-2- methyl-1,4-naphthoquinone (15b)**<br>(obtained from 6,7-dichloro-2,5,8-trihydroxy-3-methyl-1,4-naphthoquinone (**3**)):<br>Dark red solid; yield: 326 mg (55%);<br>**HRMS (ESI):** *m/z* [M-H]$^-$ calcd for $C_{13}H_{11}O_6S$: 295.0282; found: 295.0286.<br>**15a**<br>**^{1}H NMR ($CDCl_3$, 300 MHz):** δ = 13.71 (s, 1 H, α-OH), 11.67 (s, 1 H, α-OH), 6.95 (s, 1 H), 3.96 (t, *J* = 6.0 Hz, 2 H), 3.22 (t, *J* = 6.0 Hz, 2 H), 2.09 (s, 3 H).<br>**^{13}C NMR ($CDCl_3$, 75 MHz):** δ =188.3, 179.1, 158.6, 155.6, 154.5, 145.6, 120.4, 119.5, 109.0, 107.1, 60.1, 33.9, 8.4.<br><br>**15b**<br>**^{1}H NMR ($CDCl_3$, 300 MHz):** ^{1}H NMR ($CDCl_3$, 300 MHz): δ = 12.92 (s, 1 H, α-OH), 12.16 (s, 1 H, α-OH), 7.08 (s, 1 H), 3.93 (t, *J* = 6.0 Hz, 2 H), 3.21 (t, *J* = 6.0 Hz, 2 H), 2.11 (s, 3 H).<br>**^{13}C NMR ($CDCl_3$, 75 MHz):** δ =186.7, 181.2, 158.1, 156.2, 153.1, 139.8, 124.5, 122.4, 109.2, 107.6, 60.1, 33.7, 8.2.
**16**	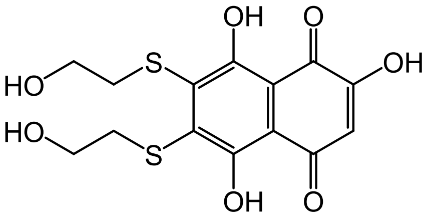	**6,7-di(2-hydroxyethylthio)-2,5,8-trihydroxy-1,4-naphthoquinone (16)** (obtained from 6,7-dichloro-2,5,8-trihydroxy-1,4-naphthoquinone (**4**)):<br>dark violet solid;<br>yield: 437 mg (61%);<br>mp 187–189 °C.<br>**IR (KBr):** 3525, 2917, 1663, 1607, 1545, 1467, 1408, 1387, 1357 cm^{-1}.<br>**^{1}H NMR (DMSO-d_6, 300 MHz):** δ = 13.91 (br s, 1 H, α-OH), 13.35 (br s, 1 H, α-OH), 12.27 (br s, 1 H, β-OH), 6.57 (s, 1 H), 5.65 (br s, 2 H, OH), 3.63-3.49 (m, 4 H), 2.75-2.63 (m, 4 H).<br>**^{13}C NMR (DMSO-d_6, 75 MHz):** δ = 186.1, 178.1, 162.3, 160.1, 158.4, 138.2, 136.5, 111.4, 110.5, 109.6, 61.5, 61.3, 36.9, 36.8.<br>**HRMS (ESI):** *m/z* [M-H]$^-$ calcd for $C_{14}H_{13}O_7S_2$: 357.0108; found: 357.0111.
**17a**<br>**17b**	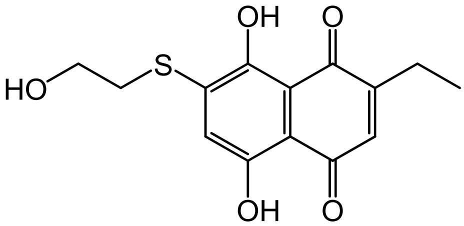	**mixture of 2-ethyl-5,8-dihydroxy-7-(2-hydroxyethylthio)-1,4-naphthoquinone (17a) and 2-ethyl-5,8-dihydroxy-6-(2-hydroxyethylthio)-1,4-naphthoquinone (17b)**<br>(obtained from 6,7-dichloro-2-ethyl-5,8-dihydroxy-1,4-naphthoquinone(**5**)):<br>red solid;<br>yield: 265 mg (45%);<br>**HRMS (ESI):** *m/z* [M-H]$^-$ calcd for $C_{14}H_{13}O_5S$: 293.0489; found: 293.0491.

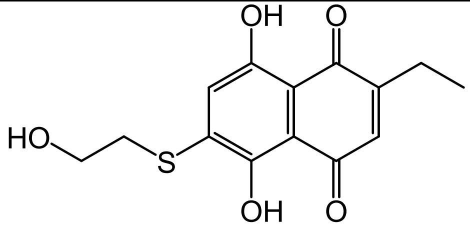

17a

^{1}H NMR ($CDCl_3$, 300 MHz): δ = 12.75 (s, 1 H, α-OH), 12.64 (s, 1 H, α-OH), 7.07 (s, 1 H), 6.73 (s, 1 H), 3.92 (t, *J* = 6.0 Hz, 2 H), 3.12 (t, *J* = 6.0 Hz, 2 H), 2.72 (q, *J* = 7.5 Hz, 2 H), 1.91 (br s, 1H, OH), 1.25 (t, *J* = 7.5 Hz, 3 H).

^{13}C NMR ($CDCl_3$, 75 MHz): δ = 180.0, 179.7, 163.1, 162.7, 152.5, 147.0, 129.5, 127.0, 110.9, 109.4, 59.9, 33.5, 23.0, 12.6.

17b

^{1}H NMR ($CDCl_3$, 300 MHz): δ = 13.06 (s, 1 H, α-OH), 12.38 (s, 1 H, α-OH), 7.02 (s, 1 H), 6.72 (s, 1 H), 3.92 (t, *J* = 6.0 Hz, 2 H), 3.12 (t, *J* = 6.0 Hz, 2 H), 2.73 (q, *J* = 7.5 Hz, 2 H), 1.91 (br s, 1H, OH), 1.25 (t, *J* = 7.5 Hz, 3 H).

^{13}C NMR ($CDCl_3$, 75 MHz): δ = 180.8, 179.4, 163.5, 161.8, 153.2, 149.4, 127.3, 126.7, 110.3, 109.9, 59.9, 33.5, 23.1, 12.6.

18a
18b

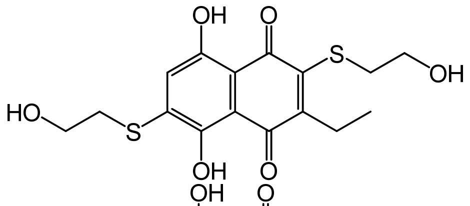

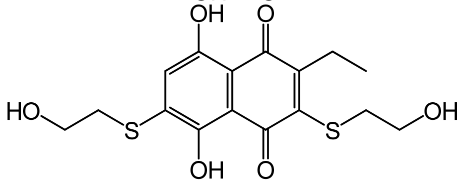

mixture of 3-ethyl-5,8-dihydroxy-2,6-di(2-hydroxyethylthio)-1,4-naphthoquinone (18a) and 2-ethyl-5,8-dihydroxy-3,6-di(2-hydroxyethylthio)-1,4-naphthoquinone (18b)
(obtained from 6,7-dichloro-2-ethyl-5,8-dihydroxy-1,4-naphthoquinone(**5**)):
red solid;
yield: 200 mg (27%);
HRMS (ESI): *m/z* $[M-H]^-$ calcd for $C_{16}H_{17}O_6S_2$: 369.0472; found: 369.0474.

18a

^{1}H NMR ($CDCl_3$, 300 MHz): δ = 13.23 (s, 1 H, α-OH), 13.08 (s, 1 H, α-OH), 6.82 (s, 1 H), 3.95 (t, *J* = 7.5 Hz, 2 H), 3.79 (t, *J* = 7.5 Hz, 2 H), 3.15 (t, *J* = 7.5 Hz, 2 H), 3.11 (t, *J* = 7.5 Hz, 2 H), 3.07 (q, *J* = 7.5 Hz, 2 H), 1.17 (t, *J* = 7.5 Hz, 3 H).

^{13}C NMR ($CDCl_3$, 75 MHz): δ = 175.6, 174.5, 167.9, 165.7, 154.2, 148.8, 136.1, 125.8, 110.1, 109.7, 60.7, 59.3, 36.1, 31.5, 22.8, 13.7.

18b

^{1}H NMR ($CDCl_3$, 300 MHz): δ = 13.36 (s, 1 H, α-OH), 13.02 (s, 1 H, α-OH), 6.81 (s, 1 H), 3.93 (t, *J* = 7.5 Hz, 2 H), 3.75 (t, *J* = 7.5 Hz, 2 H), 3.17 (t, *J* = 7.5 Hz, 2 H), 3.13 (t, *J* = 7.5 Hz, 2 H), 3.06 (q, *J* = 7.5 Hz, 2 H), 1.16 (t, *J* = 7.5 Hz, 3 H).

^{13}C NMR ($CDCl_3$, 75 MHz): δ = 175.2, 174.1, 167.3, 167.1, 151.5, 149.1, 139.4, 125.3, 110.4, 109.0, 60.6, 59.4, 36.1, 31.5, 22.8, 13.7.

19

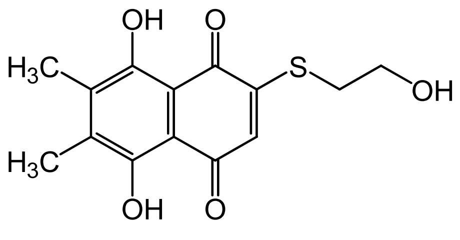

5,8-dihydroxy-2-(2-hydroxyethylthio)-6,7-dimethyl-1,4-naphthoquinone (19) (obtained from 2,3-dichloro-5,8-dihydroxy-6,7-dimethyl-1,4-naphthoquinone (**6**)):
purple solid;
yield: 194 mg (33%);
mp 133-137°C.
IR ($CHCl_3$): 3662, 3610, 2929, 2883, 2857, 1715, 1600, 1552, 1432, 1391, 1302, 1261 cm^{-1}.

^{1}H NMR ($CDCl_3$, 300 MHz): δ = 13.11 (s, 1 H, α-OH), 13.07 (s, 1 H, α-OH), 6.78 (s, 1 H), 3.97 (t, *J* = 6.0 Hz, 2 H), 3.15 (t, *J* = 6.0 Hz, 2 H), 2.25 (s, 3 H, Me), 2.24 (s, 3 H, Me).

^{13}C NMR ($CDCl_3$, 75 MHz): δ = 173.8, 172.6, 169.8, 168.5, 149.3, 141.9, 139.6, 125.1, 109.3, 108.4, 59.8,

		33.5, 12.5, 12.3.<br>**HRMS (ESI):** *m/z* [M-H]$^-$ calcd for $C_{14}H_{13}O_5S$: 293.0489; found: 293.0488.
**20**	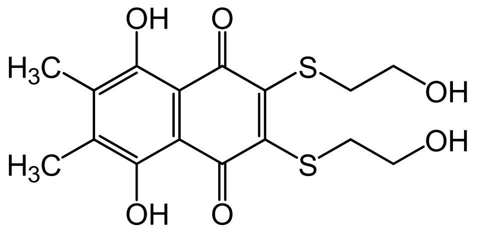	**5,8-dihydroxy-2,3-di(2-hydroxyethylthio)-6,7-dimethyl-1,4-naphthoquinone (20)** (obtained from 2,3-dichloro-5,8-dihydroxy-6,7-dimethyl-1,4-naphthoquinone (**6**)):<br>purple solid;<br>yield: 356 mg (48%);<br>mp 115-120°C.<br>**IR (CHCl$_3$):** 2932, 2875, 1601, 1395, 1330, 1297, 1269 cm^{-1}.<br>**^{1}H NMR (CDCl$_3$, 300 MHz):** δ = 13.50 (s, 2 H, 2 α-OH), 3.72 (t, *J* = 4.7 Hz, 4 H), 3.43 (t, *J* = 4.7 Hz, 4 H), 2.23 (s, 6 H, Me).<br>**^{13}C NMR (CDCl$_3$, 75 MHz):** δ = 172.7, 169.9, 144.3, 141.5, 109.6, 61.3, 38.7, 12.6.<br>**HRMS (ESI):** *m/z* [M-H]$^-$ calcd for $C_{16}H_{17}O_6S_2$: 369.0472; found: 369.0475.

Table S1.3. Synthesized compounds.

No	Formula	Spectral Information, References, Notes
**21**	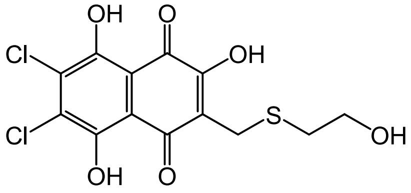	**6,7-dichloro-2,5,8-trihydroxy-3-((2-hydroxyethylthio)methyl)-1,4-naphthoquinone (21)** (obtained from 6,7-dichloro-2,5,8-trihydroxy-1,4-naphthoquinone (**4**)):<br>red solid;<br>yield: 347 mg (95%);<br>mp 165-168°C.<br>**IR (KBr):** 3415, 1608, 1402, 1277, 1217, 1178, 1135, 1114, 1054, 990 cm^{-1}.<br>**^{1}H NMR (DMSO-d$_6$, 300 MHz):** δ = 13.71 (br s, 1 H, α-OH), 13.51 (br s, 1 H, α-OH), 12.22 (br s, 1 H, β-OH), 5.62 (br s, 1 H, OH), 3.56 (s, 2 H), 3.54 (t, *J* = 6.8 Hz, 2 H), 2. 62 (t, *J* = 6.8 Hz, 2 H).<br>**^{13}C NMR (DMSO-d$_6$, 75 MHz):** δ = 186.7, 181.8, 157.5, 153.0, 152.8, 132.0, 129.7, 122.2, 110.8, 110.1, 60.9, 35.0, 23.2.<br>**HRMS (ESI):** *m/z* [M-H]$^-$ calcd for $C_{13}H_9Cl_2O_6S$: 362.9502; found: 362.9503.
**22**	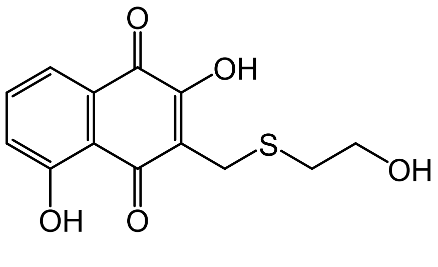	**2,5-dihydroxy-3-((2-hydroxyethylthio)methyl)-1,4-naphthoquinone (22)** (obtained from 2,5-dihydroxy-1,4-naphthoquinone (**7**)):<br>yellow solid;<br>yield: 260 mg (93%);<br>mp 124-126 °C.<br>**IR (CHCl$_3$):** 3393, 2930, 1661, 1622, 1603, 1459, 1388, 1332, 1294, 1277, 1241, 1228, 1220, 1206, 1166 cm^{-1}.<br>**^{1}H NMR (CDCl$_3$, 300 MHz):** δ = 12.28 (s, 1 H, α-OH), 7.68 (dd, *J* = 7.5, 1.0 Hz, 1 H), 7.65 (br s, 1 H, β-OH), 7.57 (t, *J* = 7.5 Hz, 1 H), 7.32 (dd, *J* = 7.5, 1.0 Hz, 1 H), 3.86 (t, *J* = 7.8 Hz, 2 H), 3.73 (s, 2 H), 2. 81 (t, *J* = 7.8 Hz, 2 H).<br>**^{13}C NMR (CDCl$_3$, 75 MHz):** δ = 189.7, 180.5, 161.6, 153.7, 135.4, 129.3, 126.8, 120.6, 119.7, 114.3, 60.8, 35.6, 22.6.<br>**HRMS (ESI):** *m/z* [M-H]$^-$ calcd for $C_{13}H_{11}O_5S$: 279.0333; found: 279.0336.

**23**	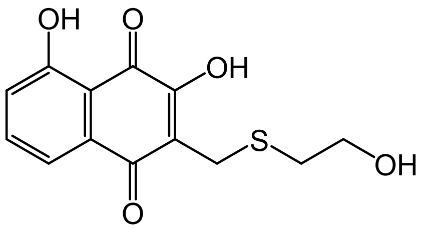	**3,5-dihydroxy-2-((2-hydroxyethylthio)methyl)-1,4-naphthoquinone (23)** (obtained from 2,8-dihydroxy-1,4-naphthoquinone (**8**)):<br>yellow solid;<br>yield: 266 mg (95%);<br>mp 132-134 °C.<br>**IR (KBr):** 3374, 2931, 1624, 1577, 1482, 1460, 1406, 1386, 1369, 1292, 1218, 1209, 1162, 1146, 1066 cm^{-1}.<br>**^{1}H NMR ($CDCl_3$, 300 MHz):** δ = 11.07 (s, 1 H, α-OH), 7.72-7.62 (m, 2 H), 7.60 (s, 1 H, β-OH), 7.23 (dd, *J* = 7.5, 2.0 Hz, 1 H), 3.85 (t, *J* = 5.6 Hz, 2 H), 3.82 (s, 2 H), 2. 80 (t, *J* = 5.6 Hz, 2 H).<br>**^{13}C NMR ($CDCl_3$, 75 MHz):** δ = 184.4, 183.0, 161.5, 153.0, 137.9, 132.3, 123.6, 122.3, 120.0, 113.0, 60.8, 35.6, 23.0.<br>**HRMS (ESI):** *m*/*z* $[M-H]^-$ calcd for $C_{13}H_{11}O_5S$: 279.0333; found: 279.0334.
**24**	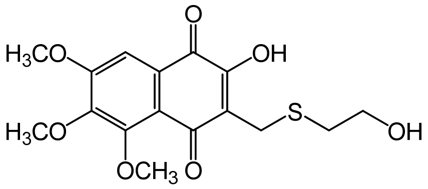	**2-hydroxy-3-((2-hydroxyethylthio)methyl)-5,6,7-trimethoxy-1,4-naphthoquinone (24)** (obtained from 2-hydroxy-5,6,7-trimethoxy-1,4-naphthoquinone (**9**)):<br>yellow solid;<br>yield: 322 mg (91%);<br>mp 126-128°C.<br>**IR (KBr):** 3413, 2941, 1659, 1574, 1484, 1468, 1454, 1416, 1373, 1328, 1251, 1215, 1200, 1142, 1100, 972 cm^{-1}.<br>**^{1}H NMR ($CDCl_3$, 300 MHz):** δ = 7.48 (s, 1 H), 7.40 (br s, 1 H, β-OH), 4.00 (s, 3 H), 3.98 (s, 3 H), 3.93 (s, 3 H), 3.84 (t, *J* = 5.8 Hz, 2 H), 3.70 (s, 2 H), 2. 81 (t, *J* = 5.8 Hz, 2 H).<br>**^{13}C NMR ($CDCl_3$, 75 MHz):** δ = 182.6, 180.7, 156.7, 154.7, 151.6, 149.7, 126.3, 122.0, 119.7, 106.2, 61.6, 61.4, 60.7, 56.4, 35.7, 23.1.<br>**HRMS (ESI):** *m*/*z* $[M-H]^-$ calcd for $C_{16}H_{17}O_7S$: 353.0700; found: 353.0703.
**25**	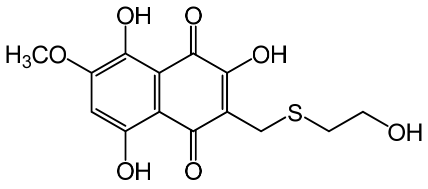	**3,5,8-trihydroxy-2-((2-hydroxyethylthio)methyl)-6-methoxy-1,4-naphthoquinone (25)** (obtained from 2,5,8-trihydroxy-7-methoxy-1,4-naphthoquinone (**10**)):<br>red solid;<br>yield: 300 mg (92%);<br>mp 195-198°C.<br>**IR (KBr):** 3530, 1594, 1484, 1455, 1437, 1408, 1385, 1363, 1309, 1195, 1064, 1024 cm^{-1}.<br>**^{1}H NMR ($CDCl_3$, 300 MHz):** δ = 13.22 (s, 1 H, α-OH), 12.03 (s, 1 H, α-OH), 7.32 (s, 1 H, β-OH), 6.59 (s, 1 H), 3.99 (s, 3 H, OCH_3), 3.87 (t, *J* = 7.8 Hz, 2 H), 3.85 (s, 2 H), 2.80 (t, *J* = 7.8 Hz, 2 H).<br>**^{13}C NMR ($CDCl_3$, 75 MHz):** δ = 181.6, 177.2, 164.2, 157.1, 155.4, 152.9, 122.8, 110.3, 109.4, 108.8, 60.8, 56.8, 35.6, 23.0.<br>**HRMS (ESI):** *m*/*z* $[M-H]^-$ calcd for $C_{14}H_{13}O_7S$: 325.0387; found: 325.0387.

Table S1.4. Synthesized compounds.

No	Formula	Spectral Information, References, Notes
**26**	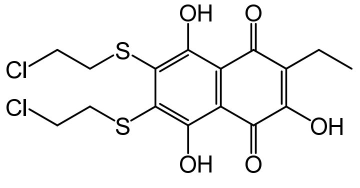	**6,7-di(2-chloroethylthio)-2-ethyl-3,5,8-trihydroxy-1,4-naphthoquinone** (obtained from 6,7-di(2-hydroxyethylthio)-2,5,8-trihydroxy-1,4-naphthoquinone (**13**)):<br>dark violet solid;<br>yield: 159 mg (75%);<br>mp 126-128°C.

		**IR ($CHCl_3$):** 3418, 2977, 1617, 1600, 1365, 1331, 1280 cm^{-1}.<br>**^1H NMR ($CDCl_3$, 300 MHz):** δ = 14.07 (s, 1 H, α-OH), 12.49 (s, 1 H, α-OH), 7.35 (s, 1 H, β-OH), 3.73-3.56 (m, 6 H), 3.50-3.43 (m, 2 H), 2.64 (q, *J* = 7.5 Hz, 2 H), 1.16 (t, *J* = 7.5 Hz, 3 H).<br>**^{13}C NMR ($CDCl_3$, 75 MHz):** δ = 187.4, 180.5, 158.0, 157.8, 153.3, 143.2, 135.4, 126.9, 109.0, 108.6, 43.3, 43.1, 36.7, 36.5, 16.3, 12.4.<br>**HRMS (ESI):** *m*/*z* $[M-H]^-$ calcd for $C_{16}H_{15}Cl_2O_5S_2$: 420.9743; found: 420.9744.
**27**	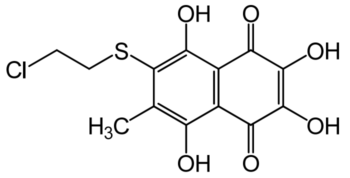	**6-(2-Chloroethylthio)-2,3,5,8-tetrahydroxy-7-methyl-1,4-naphthoquinone** (obtained from 2,3,5,8-tetrahydroxy-6-(2-hydroxyethylthio)-7-methyl -1,4-naphthoquinone (**14**)):<br>purple solid;<br>yield: 136 mg (82%);<br>mp 162-166°C.<br>**IR ($CHCl_3$):** 3662, 3433, 1693, 1599, 1416, 1376, 1310, 1288 cm^{-1}.<br>**^1H NMR ($CDCl_3$, 300 MHz):** δ = 12.69 (s, 1 H, α-OH), 12.23 (s, 1 H, α-OH), 6.70 (s, 2 H, β-OH), 3.60 (t, *J* = 7.5 Hz, 2 H), 3 41 (t, *J* = 7.5 Hz, 2 H), 2.54 (s, 3 H).<br>**^{13}C NMR (DMSO-d_6, 75 MHz):** δ = 183.3, 183.1, 156.5, 154.5, 142.3, 141.2, 141.2, 132.8, 108.0, 106.9, 43.9, 35.7, 14.5.<br>**HRMS (ESI):** *m*/*z* $[M-H]^-$ calcd for $C_{13}H_{10}ClO_6S$: 328.9892; found: 328.9896.
**28**	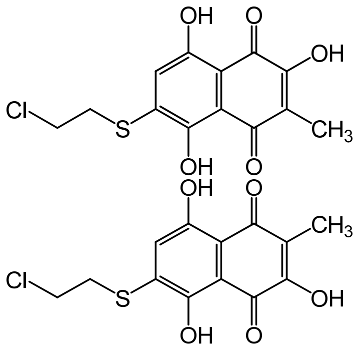	**mixture of 6-(2-chloroethylthio)-2,5,8-trihydroxy-3-methyl-1,4-naphthoquinone (28a) and 6-(2-chloroethylthio)-3,5,8-trihydroxy-2-methyl-1,4-naphthoquinone (28b)**<br>(obtained from the mixture of 2,5,8-trihydroxy-6-(2-hydroxyethylthio)-3-methyl-1,4-naphthoquinone (**15a**) and 3,5,8-trihydroxy-6-(2-hydroxyethylthio)-2- methyl-1,4-naphthoquinone (**15b**)):<br>dark red solid;<br>yield: 134 mg (85%);<br>**HRMS (ESI):** *m*/*z* $[M-H]^-$ calcd for $C_{13}H_{10}ClO_5S$: 312.9943; found: 312.9945.<br>**6-(2-chloroethylthio)-2,5,8-trihydroxy-3-methyl-1,4-naphthoquinone (28a)**<br>^1H NMR ($CDCl_3$, 700 MHz): δ = 13.64 (s, 1 H, α-OH), 11.61 (s, 1 H, α-OH), 7.45 (s, 1 H, β-OH), 6.88 (s, 1 H), 3.75 (t, *J* = 7.5 Hz, 2 H), 3.37 (t, *J* = 7.5 Hz, 2 H), 2.08 (s, 3 H).<br>^{13}C NMR ($CDCl_3$, 175 MHz): δ = 188.3, 179.3, 158.4, 155.4, 154.5, 144.3, 120.6, 119.4, 109.2, 107.4, 40.9, 32.7, 8.1.<br>**6-(2-chloroethylthio)-3,5,8-trihydroxy-2-methyl-1,4-naphthoquinone (28b)**<br>^1H NMR ($CDCl_3$, 700 MHz): δ = 12.87 (s, 1 H, α-OH), 12.10 (s, 1 H, α-OH), 7.25 (s, 1 H, β-OH), 7.03 (s, 1 H), 3.73 (t, *J* = 7.5 Hz, 2 H), 3.36 (t, *J* = 7.5 Hz, 2 H), 2.10 (s, 3 H).<br>^{13}C NMR ($CDCl_3$, 175 MHz): δ = 186.9, 181.3, 157.9, 155.9, 153.1, 138.5, 124.5, 122.4, 109.4, 107.9, 41.1, 32.9, 8.3.

**29**	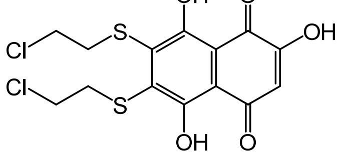	**6,7-di(2-chloroethylthio)-2,5,8-trihydroxy-1,4-naphthoquinone** (obtained from 6,7-di(2-hydroxyethylthio)-2,5,8-trihydroxy-1,4-naphthoquinone (**16**)):<br>purple solid;<br>yield: 134 mg (68%);<br>mp 133–136 °C.<br>**IR ($CHCl_3$):** 3518, 3413, 2953, 1659, 1605, 1534, 1442, 1403, 1384, 1343, 1269, 1194, 1106 cm^{-1}.<br>**^{1}H NMR ($CDCl_3$, 300 MHz):** δ = 13.84 (s, 1 H, α-OH), 12.52 (s, 1 H, α-OH), 7.41 (s, 1 H, β-OH), 6.43 (s, 1 H), 3.75-3.61 (m, 6 H), 3. 48 (t, *J* = 7.5 Hz, 2 H).<br>**^{13}C NMR ($CDCl_3$, 75 MHz):** δ = 185.6, 177.2, 160.8, 159.8, 157.0, 137.9, 135.8, 111.0, 109.1, 108.7, 43.5, 43.3, 36.9, 36.5.<br>**HRMS (ESI):** *m*/*z* $[M-H]^-$ calcd for $C_{14}H_{11}Cl_2O_5S_2$: 392.9430; found: 392.9431.
**30**	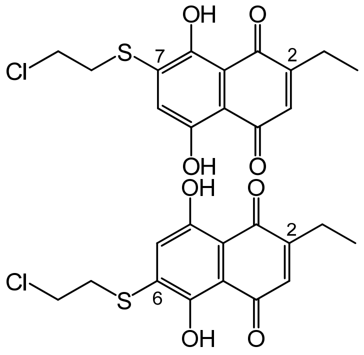	**Equimolar mixture of 7-(2-chloroethylthio)-2-ethyl-5,8-dihydroxy-1,4-naphthoquinone (30a) and 6-(2-chloroethylthio)-2-ethyl-5,8-dihydroxy-1,4-naphthoquinone (30b)**<br>(obtained from the mixture of 2-ethyl-5,8-dihydroxy-7-(2-hydroxyethylthio)-1,4-naphthoquinone (**17a**) and 2-ethyl-5,8-dihydroxy-6-(2-hydroxyethylthio)-1,4-naphthoquinone (**17b**)):<br>red solid; yield:<br>134 mg (86%);<br>**HRMS (ESI):** *m*/*z* $[M-H]^-$ calcd for $C_{14}H_{12}ClO_4S$: 311.0150; found: 311.0151.<br>**7-(2-chloroethylthio)-2-ethyl-5,8-dihydroxy-1,4-naphthoquinone (30a)**<br>**^{1}H NMR ($CDCl_3$, 300 MHz):** δ = 12.73 (s, 1 H, α-OH), 12.62 (s, 1 H, α-OH), 7.07 (s, 1 H), 6.68 (s, 1 H), 3.76 (t, *J* = 7.5 Hz, 2 H), 3.28 (t, *J* = 7.5 Hz, 2 H), 2.71 (q, *J* = 7.5 Hz, 2 H), 1.25 (t, *J* = 7.5 Hz, 3 H).<br>**^{13}C NMR ($CDCl_3$, 75 MHz):** δ = 179.1, 179.0, 163.8, 163.3, 151.2, 147.3, 129.6, 126.6, 110.8, 109.3, 40.4, 32.4, 22.9, 12.5<br>**6-(2-chloroethylthio)-2-ethyl-5,8-dihydroxy-1,4-naphthoquinone (30b)**<br>**^{1}H NMR ($CDCl_3$, 300 MHz):** δ = 13.03 (s, 1 H, α-OH), 12.36 (s, 1 H, α-OH), 7.01 (s, 1 H), 6.66 (s, 1 H), 3.76 (t, *J* = 7.5 Hz, 2 H), 3.28 (t, *J* = 7.5 Hz, 2 H), 2.73 (q, *J* = 7.5 Hz, 2 H), 1.26 (t, *J* = 7.5 Hz, 3 H).<br>**^{13}C NMR ($CDCl_3$, 75 MHz):** δ = 180.1, 178.5, 164.1, 162.4, 152.0, 149.6, 127.5, 126.2, 110.1, 109.9, 40.4, 32.4, 23.1, 12.5.
**31**	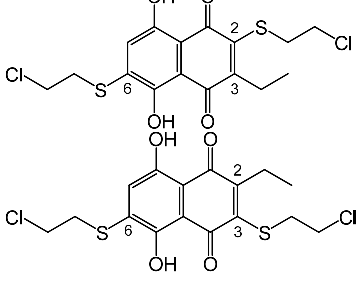	**Equimolar mixture of 2,6-di(2-chloroethylthio)-3-ethyl-5,8-dihydroxy-1,4-naphthoquinone (31a) and 3,6-di(2-chloroethylthio)-2-ethyl-5,8-dihydroxy-1,4-naphthoquinone (31b)**<br>(obtained from the mixture of 3-ethyl-5,8-dihydroxy-2,6-di(2-hydroxyethylthio)-1,4-naphthoquinone (**18a**) and 2-ethyl-5,8-dihydroxy-3,6-di(2-hydroxyethylthio)-1,4-naphthoquinone (**18b**)):<br>dark red solid;<br>yield: 141 mg (69%);<br>**HRMS (ESI):** *m*/*z* $[M-H]^-$ calcd for $C_{16}H_{15}Cl_2O_4S_2$: 404.9794; found: 404.9792.<br>**2,6-di(2-chloroethylthio)-3-ethyl-5,8-dihydroxy-**

		**1,4-naphthoquinone (31a)**<br>**^{1}H NMR ($CDCl_3$, 300 MHz):** δ = 13.21 (s, 1 H, α-OH), 13.06 (s, 1 H, α-OH), 6.77 (s, 1 H), 3.76 (t, *J* = 7.5 Hz, 2 H), 3.65 (t, *J* = 7.5 Hz, 2 H), 3.45 (t, *J* = 7.5 Hz, 2 H), 3.31 (t, *J* = 7.5 Hz, 2 H), 3.06 (q, *J* = 7.5 Hz, 2 H), 1.17 (t, *J* = 7.5 Hz, 3 H).<br>**^{13}C NMR ($CDCl_3$, 75 MHz):** δ = 174.8, 173.8, 168.6, 166.2, 153.3, 149.1, 136.8, 125.4, 109.9, 109.6, 43.4, 40.5, 36.0, 32.5, 22.6, 13.5.<br>**3,6-di(2-chloroethylthio)-2-ethyl-5,8-dihydroxy-1,4-naphthoquinone (31b)**<br>**^{1}H NMR ($CDCl_3$, 300 MHz):** δ = 13.34 (s, 1 H, α-OH), 13.00 (s, 1 H, α-OH), 6.76 (s, 1 H), 3.76 (t, *J* = 7.5 Hz, 2 H), 3.65 (t, *J* = 7.5 Hz, 2 H), 3.51 (t, *J* = 7.5 Hz, 2 H), 3.31 (t, *J* = 7.5 Hz, 2 H), 3.03 (q, *J* = 7.5 Hz, 2 H), 1.16 (t, *J* = 7.5 Hz, 3 H).<br>**^{13}C NMR ($CDCl_3$, 75 MHz):** δ = 174.5, 173.2, 167.9, 167.7, 150.4, 149.3, 139.6, 124.8, 110.3, 108.9, 43.5, 40.5, 36.0, 32.5, 22.4, 13.3.
**32**	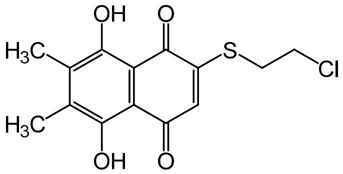	**2-(2-Chloroethylthio)-5,8-dihydroxy-6,7-dimethyl-1,4-naphthoquinone** (obtained from 5,8-dihydroxy-2-(2-hydroxyethylthio)-6,7-dimethyl-1,4-naphthoquinone (**19**)):<br>purple solid;<br>yield: 120 mg (77%);<br>mp 125-127°C.<br>**IR ($CHCl_3$):** 2927, 2856, 1733, 1715, 1601, 1555, 1457, 1391, 1300, 1267 cm^{-1}.<br>**^{1}H NMR ($CDCl_3$, 300 MHz):** δ = 13.09 (s, 1 H, α-OH), 13.07 (s, 1 H, α-OH), 6.79 (s, 1 H), 3.75 (t, *J* = 7.5 Hz, 2 H), 3.31 (t, *J* = 7.5 Hz, 2 H), 2.54 (s, 3 H).<br>**^{13}C NMR ($CDCl_3$, 75 MHz):** δ = 172.6, 171.5, 171.1, 170.3, 147.8, 142.5, 140.3, 124.8, 109.7, 108.7, 40.8, 32.6, 12.7, 12.5.<br>**HRMS (ESI):** *m/z* $[M-H]^-$ calcd for $C_{14}H_{12}ClO_4S$: 311.0150; found: 311.0152.
**33**	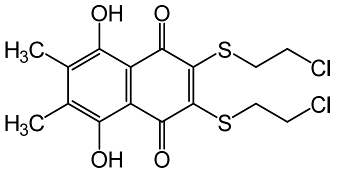	**2,3-di(2-chloroethylthio)-5,8-dihydroxy-6,7-dimethyl-1,4-naphthoquinone** (obtained from 5,8-dihydroxy-2,3-di(2-hydroxyethylthio)-6,7-dimethyl-1,4-naphthoquinone (**20**)):<br>purple solid;<br>yield: 132 mg (65%);<br>mp 132-136°C.<br>**IR ($CHCl_3$):** 2928, 2856, 1600, 1445, 1392, 1379, 1297, 1270 cm^{-1}.<br>**^{1}H NMR ($CDCl_3$, 300 MHz):** δ = 13.51 (s, 2 H, 2 α-OH), 3.72 (t, *J* = 7.4 Hz, 4 H), 3.59 (t, *J* = 7.4 Hz, 4 H), 2.26 (s, 6 H, Me).<br>**^{13}C NMR ($CDCl_3$, 75 MHz):** δ = 171.9, 170.1, 143.0, 141.3, 109.4, 43.5, 36.6, 12.5.<br>**HRMS (ESI):** *m/z* $[M-H]^-$ calcd for $C_{16}H_{15}Cl_2O_4S_2$: 406.3246; found: 406.3250.
**34**	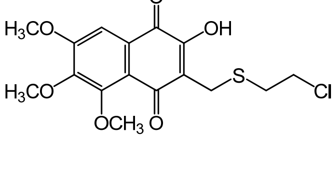	**3-((2-chloroethylthio)methyl)-2-hydroxy-5,6,7-trimethoxy-1,4-naphthoquinone** (obtained from 2-hydroxy-3-((2-hydroxyethylthio)methyl)-5,6,7-trimethoxy-1,4-naphthoquinone (**24**)):<br>yellow solid;<br>yield: 158 mg (85%);<br>mp 129-132°C.<br>**IR ($CHCl_3$):** 3410, 3001, 1704, 1655, 1578, 1483, 1466, 1418, 1374, 1334, 1294, 1143,1102 cm^{-1}.<br>**^{1}H NMR ($CDCl_3$, 300 MHz):** δ = 7.48 (s, 1 H), 7.28 (br s, 1 H, β-OH), 4.00 (s, 3 H), 3.98 (s, 3 H), 3.93

		(s, 3 H), 3.72 (t, *J* = 7.8 Hz, 2 H), 3.69 (s, 2 H), 2. 92 (t, *J* = 7.8 Hz, 2 H).<br>**^{13}C NMR (CDCl$_3$, 75 MHz):** δ = 182.3, 180.7, 156.7, 154.8, 151.5, 149.8, 126.2, 121.8, 119.7, 106.2, 61.7, 61.4, 56.5, 42.9, 34.4, 23.5.<br>**HRMS (ESI):** *m*/*z* [M-H]$^-$ calcd for $C_{16}H_{17}ClO_6S$: 371.0361; found: 371.0364.
**35**	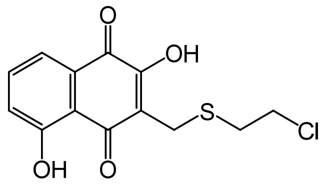	**3-((2-chloroethylthio)methyl)-2,5-dihydroxy-1,4-naphthoquinone** (obtained from 2,5-dihydroxy-3-((2-hydroxyethylthio)methyl)-1,4-naphthoquinone (**22**)):<br>yellow solid;<br>yield: 114 mg (76%);<br>mp 116-118 °C.<br>**IR (CHCl$_3$):** 3391, 3010, 1660, 1623, 1459, 1415, 1387, 1332, 1294, 1277, 1191, 1167,1070 cm^{-1}.<br>**^{1}H NMR (CDCl$_3$, 300 MHz):** δ = 12.27 (s, 1 H, α-OH), 7.68 (dd, *J* = 7.5, 1.0 Hz, 1 H), 7.65 (br s, 1 H, β-OH), 7.57 (t, *J* = 7.5 Hz, 1 H), 7.32 (dd, *J* = 7.5, 1.0 Hz, 1 H), 3.73 (t, *J* = 7.8 Hz, 2 H), 3.71 (s, 2 H), 2. 93 (t, *J* = 7.8 Hz, 2 H).<br>**^{13}C NMR (CDCl$_3$, 75 MHz):** δ = 189.6, 180.3, 161.6, 153.6, 135.4, 129.3, 126.7, 120.6, 119.7, 114.2, 42.8, 34.4, 22.9.<br>**HRMS (ESI):** *m*/*z* [M-H]$^-$ calcd for $C_{13}H_{10}ClO_4S$: 296.9996; found: 296.9994.
**36**	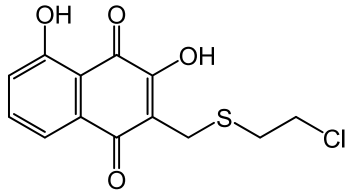	**2-((2-chloroethylthio)methyl)-3,5-dihydroxy-1,4-naphthoquinone** (obtained from 3,5-dihydroxy-2-((2-hydroxyethylthio)methyl)-1,4-naphthoquinone (**23**)):<br>yellow solid;<br>yield: 117 mg (78%);<br>mp 119-121 °C.<br>**IR (CHCl$_3$):** 3412, 3003, 1653, 1630, 1577, 1461, 1417, 1367, 1318, 1277, 1170 cm^{-1}.<br>**^{1}H NMR (CDCl$_3$, 500 MHz):** δ = 11.05 (s, 1 H, α-OH), 7.70-7.64 (m, 2 H), 7.52 (s, 1 H, β-OH), 7.23 (dd, *J* = 8.0, 1.5 Hz, 1 H), 3.72 (t, *J* = 7.6 Hz, 2 H), 3.71 (s, 2 H), 2. 93 (t, *J* = 7.6 Hz, 2 H).<br>**^{13}C NMR (CDCl$_3$, 125 MHz):** δ = 184.3, 182.8, 161.5, 152.8, 138.0, 132.3, 123.6, 122.1, 120.0, 113.0, 42.8, 34.4, 23.4.<br>**HRMS (ESI):** *m*/*z* [M-H]$^-$ calcd for $C_{13}H_{10}ClO_4S$: 296.9994; found: 296.9994.
**37**	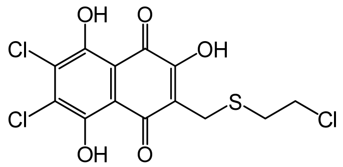	**6,7-dichloro-2-((2-chloroethylthio)methyl)-3,5,8-trihydroxy-1,4-naphthoquinone** (obtained from 6,7-dichloro-2,5,8-trihydroxy-3-((2-hydroxyethylthio)-methyl)-1,4-naphthoquinone (**21**)):<br>red solid;<br>yield: 139 mg (72%);<br>mp 143-147 °C.<br>**IR (CHCl$_3$):** 3410, 3010, 1657, 1630, 1610, 1553, 1432, 1402, 1323, 1271, 1226, 1212, 1204, 1180, 1113 cm^{-1}.<br>**^{1}H NMR (CDCl$_3$, 500 MHz):** δ = 13.34 (s, 1 H, α-OH), 12.01 (s, 1 H, α-OH), 7.55 (s, 1 H, β-OH), 3.74 (s, 2 H), 3.72 (t, *J* = 7.8 Hz, 2 H), 2. 94 (t, *J* = 7.8 Hz, 2 H).<br>**^{13}C NMR (CDCl$_3$, 125 MHz):** δ = 186.3, 180.7, 154.7 (2C), 153.7, 135.7, 131.7, 122.5, 108.9, 108.8, 42.7, 34.6, 23.0.<br>**HRMS (ESI):** *m*/*z* [M-H]$^-$ calcd for $C_{13}H_8Cl_3O_5S$: 380.9163; found: 380.9165.

**38**	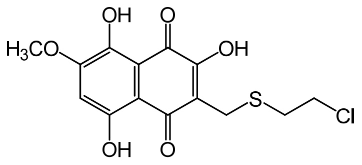	**2-((2-chloroethylthio)methyl)-3,5,8-trihydroxy-6-methoxy-1,4-naphthoquinone** (obtained from 3,5,8-trihydroxy-2-((2-hydroxyethylthio)methyl)-6-methoxy-1,4-naphthoquinone (**25**)):<br>red solid;<br>yield: 128 mg (74%);<br>mp 176-179 °C.<br>**IR ($CHCl_3$):** 3523, 3411, 2942, 1602, 1478, 1436, 1413, 1396, 1354, 1323, 1304, 1274, 1217, 1210, 1196, 1178 cm^{-1}.<br>**^1H NMR ($CDCl_3$, 300 MHz):** δ = 13.20 (s, 1 H, α-OH), 12.02 (s, 1 H, α-OH), 7.30 (s, 1 H, β-OH), 6.59 (s, 1 H), 3.98 (s, 3 H, OCH_3), 3.75 (s, 2 H), 3.73 (t, J = 7.8 Hz, 2 H), 2. 93 (t, J = 7.8 Hz, 2 H).<br>**^{13}C NMR ($CDCl_3$, 75 MHz):** δ = 181.5, 177.1, 164.0, 157.0, 155.4, 152.9, 122.8, 110.3, 109.4, 108.8, 56.7, 42.8, 34.4, 23.1.<br>**HRMS (ESI):** m/z $[M-H]^-$ calcd for $C_{14}H_{12}ClO_6S$: 343.0048; found: 343.0051.

BIOLOGY

Cell culture conditions

Cells were incubated at 37°C in a humidified atmosphere with 5% (v/v) CO_2. Cells were continuously kept in culture for a maximum of 3 months, and were routinely inspected microscopically for stable phenotype and regularly checked for contamination with mycoplasma.

PC-3, DU145, LNCaP, 22Rv1 and PNT2 cells were cultured in 10% FBS/RPMI medium (RPMI medium supplemented with Glutamax™-I (Invitrogen, Paisley, UK) containing 10% fetal bovine serum (FBS, Invitrogen) and 1% penicillin/streptomycin (Invitrogen)). MRC-9, HEK 293 and VCaP cells were cultured in 10% FBS/DMEM medium (DMEM medium supplemented with Glutamax™-I (Invitrogen) containing 10% FBS and 1% penicillin/streptomycin (Invitrogen)). RWPE-1 cells were cultured in Clonetics® EGM™-2 SingleQuots® medium (Lonza, Walkersville, MD, USA) containing 10% FBS. HUVEC cells (passage 11) were cultured in Clonetics® EGM™-2 SingleQuots® medium (Lonza, Walkersville, MD, USA) containing 10% FBS.

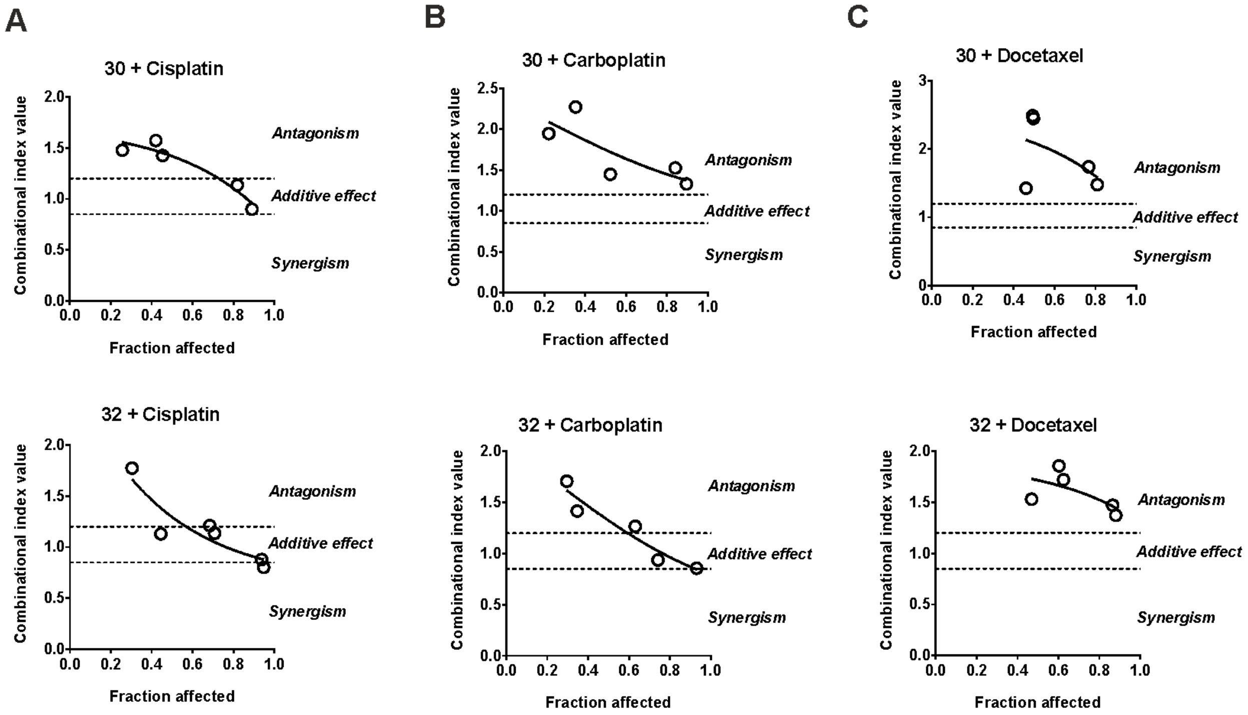

Figure S1. Effects of **30** and **32** on cell viability in combination with platinum or taxane agents.

22Rv1 cells were co-treated with indicated drug combinations for 48 h and the viability was evaluated using MTT viability assay and analyzed using Chou-Talalay method. Combinational index (CI) values were calculated using CompuSyn 1.0 software.

Table S2. Molar ratios of the individual drugs used for drug combinational studies.

Figure 3H	**30** : Olaparib	0.6 : 100
	**32** : Olaparib	0.4 : 100
Figure 4A	**30** : SB203580	0.5 : 200
	**30** : SCH772984	0.5 : 4
	**30** : PD98059	0.5 : 200
	**30** : GSK-690396	0.5 : 40
	**30** : GSK-SP600125	0.5 : 100
Figure 4C	**32** : SB203580	0.5 : 200
	**32** : SCH772984	0.5 : 4
	**32** : PD98059	0.5 : 200
	**32** : GSK-690396	0.5 : 40
	**32** : GSK-SP600125	0.5 : 100
Figure 5B	**30** : Enzalutamide	0.6 : 100
	**32** : Enzalutamide	0.4 : 100
Figure S1	**30**: Cisplatin	0.6 : 10
	**30**: Carboplatin	0.6 : 150
	**30**: Docetaxel	0.6 : 0.02
	**32** : Cisplatin	0.4 : 10
	**32** : Carboplatin	0.4 : 150
	**32** : Docetaxel	0.4 : 0.02
